# Supplementary material for: Comparison of nebivolol versus diltiazem in improving coronary artery spasm and quality of life in patients with hypertension and vasospastic angina: A prospective, randomized, double-blind pilot study
Source: PLoS One. 2020 Sep 11;15(9):e0239039. doi: 10.1371/journal.pone.0239039 (PMC7485806; doi:10.1371/journal.pone.0239039)
Supplement: S1 Table — (DOCX) [file pone.0239039.s001.docx]

**S1 Table**

| **Baseline** | **Nebivolol group (n=15)** | **Diltiazem group (n=16)** | **Combination group (n=17)** | **p-value** |
| --- | --- | --- | --- | --- |
| **Physical limitation** | | | | |
| **Score Sum** | 40.7±6.6 | 42.5±5.1 | 40.0±5.6 | 0.455 |
| **Percentile** | 47.4±27.3 | 47.7±23.9 | 39.7±27.0 | 0.611 |
| **Category** | 2.47±1.06 | 2.50±0.97 | 2.82±1.01 | 0.542 |
| **Angina frequency** | | | | |
| **Score Sum** | 8.5±2.0 | 8.8±2.0 | 9.1±1.7 | 0.633 |
| **Percentile** | 39.6±29.7 | 42.7±27.3 | 50.6±25.4 | 0.506 |
| **Category** | 2.80±1.21 | 2.63±1.15 | 2.24±1.15 | 0.378 |
| **Quality of life** | | | | |
| **Score Sum** | 11.4±4.7 | 10.4±3.2 | 10.9±3.2 | 0.774 |
| **Percentile** | 52.5±33.8 | 47.7±28.9 | 52.1±26.2 | 0.878 |
| **Category** | 2.87±1.41 | 3.13±0.96 | 2.77±1.15 | 0.670 |
| **Anginal stability** | | | | |
| **Score Sum** | 3.9±1.6 | 2.8±1.3 | 3.1±1.3 | 0.100 |
| **Percentile** | 54.7±31.3 | 35.1±25.0 | 38.1±25.5 | 0.110 |
| **Category** | 2.73±1.75 | 3.81±1.33 | 3.65±1.41 | 0.111 |
| **Treatment Satisfaction** | | | | |
| **Score Sum** | 16.5±2.4 | 16.1±1.6 | 16.4±2.6 | 0.829 |
| **Percentile** | 44.7±31.1 | 37.7±21.6 | 44.2±25.9 | 0.704 |
| **Category** | 2.67±1.18 | 2.88±0.96 | 2.59±0.94 | 0.713 |
| **Follow-up** | **Nebivolol group (n=14)** | **Diltiazem group (n=14)** | **Combination group (n=17)** | **p-value** |
| **Physical limitation** | | | | |
| **Score Sum** | 44.8±3.6 | 42.0±7.5 | 45.2±4.2 | 0.215 |
| **Percentile** | 49.1±25.6 | 36.2±22.4 | 52.3±25.2 | 0.178 |
| **Category** | 2.50±0.85 | 3.00±0.78 | 2.47±0.94 | 0.194 |
| **Angina frequency** | | | | |
| **Score Sum** | 10.7±0.7 | 9.8±1.6 | 10.0±1.5 | 0.167 |
| **Percentile** | 46.1±27.6 | 31.2±24.2 | 37.1±21.6 | 0.278 |
| **Category** | 2.86±1.10 | 3.43±0.85 | 3.06±0.90 | 0.283 |
| **Quality of life** | | | | |
| **Score Sum** | 10.8±1.7 | 10.5±1.9 | 10.3±2.3 | 0.793 |
| **Percentile** | 46.1±28.9 | 42.0±32.6 | 42.0±31.0 | 0.921 |
| **Category** | 2.64±1.08 | 2.86±1.23 | 2.77±1.15 | 0.886 |
| **Anginal stability** | | | | |
| **Score Sum** | 4.8±0.6 | 4.3±0.6 | 4.4±0.7 | 0.111 |
|  |  |  |  |  |
| **Percentile** | 42.8±25.2 | 24.3±21.3 | 31.2±22.3 | 0.110 |
| **Category** | 3.29±1.27 | 4.21±1.12 | 3.77±1.25 | 0.143 |
| **Treatment Satisfaction** | | | | |
| **Score Sum** | 15.9±1.5 | 15.9±2.4 | 16.7±2.0 | 0.419 |
| **Percentile** | 36.7±21.4 | 42.4±32.6 | 49.1±29.2 | 0.479 |
| **Category** | 2.93±1.00 | 2.64±1.34 | 2.53±1.18 | 0.637 |

Values are presented as number of patients (%) or mean±standard deviation.
