# Supplementary material for: Comparison of nebivolol versus diltiazem in improving coronary artery spasm and quality of life in patients with hypertension and vasospastic angina: A prospective, randomized, double-blind pilot study
Source: PLoS One. 2020 Sep 11;15(9):e0239039. doi: 10.1371/journal.pone.0239039 (PMC7485806; doi:10.1371/journal.pone.0239039)
Supplement: S2 Table — (DOCX) [file pone.0239039.s002.docx]

**S2 Table**

|  | **Nebivolol group (n=15)** | **Diltiazem group (n=16)** | **Combination group (n=17)** | **p-value** |
| --- | --- | --- | --- | --- |
| **Visit 1** |  |  |  |  |
| **SBP** | 132.1±18.5 | 136.2±15.3 | 137.9±15.7 | 0.602 |
| **DBP** | 76.3±9.1 | 81.9±8.9 | 83.7±12.5 | 0.124 |
| **Pulse pressure** | 55.9±18.2 | 54.3±16.1 | 54.2±11.3 | 0.946 |
| **Visit 2** |  |  |  |  |
| **SBP** | 137.0±18.8 | 135.6±12.9 | 140.6±15.7 | 0.647 |
| **DBP** | 77.8±9.4 | 82.6±7.9 | 85.8±12.0 | 0.090 |
| **Pulse pressure** | 59.2±15.1 | 53.0±16.7 | 54.8±12.2 | 0.491 |
| **Visit 3** |  |  |  |  |
| **SBP** | 128.4±16.4 | 129.3±19.4 | 129.9±12.0 | 0.964 |
| **DBP** | 77.7±10.4 | 80.0±8.7 | 83.3±11.3 | 0.305 |
| **Pulse pressure** | 50.7±10.2 | 49.3±13.5 | 49.6±11.9 | 0.621 |
| **Visit 4** |  |  |  |  |
| **SBP** | 128.6±17.6 | 121.7±13.8 | 123.5±11.8 | 0.429 |
| **DBP** | 75.6±10.7 | 75.2±8.6 | 79.6±11.9 | 0.451 |
| **Pulse pressure** | 53.1±12.2 | 46.5±8.8 | 43.9±7.4 | 0.034 |
| **Visit 5** |  |  |  |  |
| **SBP** | 124.2±20.5 | 121.9±15.4 | 125.4±17.8 | 0.859 |
| **DBP** | 66.1±12.4 | 76.2±8.5 | 71.5±10.9 | 0.054 |
| **Pulse pressure** | 58.1±18.0 | 45.6±11.8 | 53.9±18.1 | 0.131 |

Values are presented as number of patients (%) or mean±standard deviation. SBP, systolic blood pressure; DBP, diastolic blood pressure.
