# Supplementary material for: Comparison of nebivolol versus diltiazem in improving coronary artery spasm and quality of life in patients with hypertension and vasospastic angina: A prospective, randomized, double-blind pilot study
Source: PLoS One. 2020 Sep 11;15(9):e0239039. doi: 10.1371/journal.pone.0239039 (PMC7485806; doi:10.1371/journal.pone.0239039)
Supplement: S2 File — (DOCX) [file pone.0239039.s004.docx]

|  | **임상시험계획서** |
| --- | --- |

| **혈관 수축성 협심증을 동반한 고혈압 환자에서**  **네비보롤(Nebivolol)의 효과에 대한 예비(pilot) 시험**  (연구자주도 임상시험) |
| --- |

**임상시험계획서 NO. NEB-VAS-01**

**Version 1.5**

| 본 임상시험계획서에 포함된 모든 정보는 임상시험책임자 및 임상시험담당자, 임상시험심사위원회에 제공된 것으로서, 임상시험에 사용되는 의약품을 투여 받는 사람에게 시험 참가에 대한 서면 동의를 받기 위한 경우를 제외 하고는 사전 서면 동의 없이 제3자에게 공개될 수 없습니다. |
| --- |

**[임상시험계획서 개요]**

| **임상시험**  **제목** | 혈관 수축성 협심증을 동반한 고혈압 환자에서 네비보롤(Nebivolol)의 효과에 대한 예비(pilot) 시험  A pilot study for the Effect of Nebivolol on Coronary Arterial Spasm in Patients with Hypertension |
| --- | --- |
| **임상시험용**  **의약품** | 네비레트정(네비보롤염산염)  헤르벤서방정(딜티아젬염산염) |
| **목 적** | 혈관 수축성 협심증을 동반한 고혈압 환자에서 네비보롤 단독, 딜티아젬 단독 또는 저용량 네비보롤/딜티아젬 병용요법 간의 관동맥 연축과 혈압 강하에 대한 효과 확인 |
| **시험디자인** | 전향적, 무작위배정, 이중눈가림, 연구자주도, 예비(Pilot) 임상시험 |
| **대상질환** | 혈관 수축성 협심증을 동반한 고혈압 환자 |
| **시험대상자수** | 45명 (각 군당 15명) |
| **선정/제외**  **기준** | 선정기준  1) 만 20세~80세의 고혈압(stage I-2: 수축기 혈압 140-179mmHg 및 이완기 혈압 90-109mmHg) 환자들 중 관상동맥조영술 및 혈관수축유발 검사를 통해 혈관 수축성 협심증을 진단 받은 환자로 통원치료가 가능한 자  2) 본 임상시험에 참여를 결정하며 자발적으로 동의서에 서명한 자  제외기준  1) 베타 차단제 혹은 칼슘 차단제에 과민 반응을 보인 기왕력  2) 치매 또는 동반된 정신과 질환이 있거나 약물 오남용의 기왕력  3) 스크리닝 전 1개월 이내에 다른 임상시험에 참여한 자  4) 계획서의 준수사항과 진행 절차를 이행할 수 없거나 참여에 부적합한 의학적 상태로 시험자가 판단한 자  5) 연구약물의 약효평가에 영향을 줄 수 있는 약(ACE 억제제, 안지오텐신차단제, 임상시험용의약품외 베타차단제, 임상시험용의약품외 칼슘길항제, 인다파마이드를 제외한 이뇨제)을 복용하고 있는 시험대상자 (이러한 시험대상자는 최소한 2주간의 wash-out period 후 참여 가능)  6) 악성 고혈압 (망막 출혈 혹은 유두 부종이 있는 경우) 혹은 알려진 중등증 또는 악성 망막증 (최근 6개월 이내 망막 출혈, 시력 장애, 망막 미세동맥류)  7) 이차성 고혈압의 과거력 및 이차성 고혈압이 의심되는 모든 병력: 대동맥축착증, 고알도스테론혈증, 신동맥협착, 쿠싱병, 크롬친화세포종, 다낭성 신질환 등  8) 증상을 동반한 기립성 저혈압 환자  9) 중증의 심장 질환자 (심부전(NYHA class 3와 4), 최근 6개월 이내 허혈성 심장질환 (협심증, 심근경색), 경피적 관상동맥 확장술, 또는 관상동맥 우회술 치료 등을 받은 자)  10) 중증의 뇌혈관장애 환자 (최근 6개월 이내 뇌졸중, 뇌경색, 뇌출혈 등)  11) 무뇨 또는 중증의 신부전 환자 (creatinine clearance < 30mL/min)  12) 중증 간부전이거나 AST 또는 ALT > 정상 상한치의 3배, 담도 폐쇄 담즙성 간경변, 담즙 정체 환자  13) 약물의 흡수, 분포, 대사, 배설에 영향을 줄 수 있는 위장관 질환 및 수술 환자, 시험자가 임상적으로 유의하다고 판단하는 현재의 활동성 위염 및 위장관/직장 출혈, 최근 12개월 이내의 활동성 염증성 대장 증후군 등.  14) 임부 및 수유부, 임상시험 기간 동안 임신 계획이 있거나 적절한 피임방법에 동의하지 않는 가임 여성  * 적절한 피임방법은 다음과 같다.  (1) Progestin 단일제의 호르몬 요법 (경구, 이식정)  (2) 자궁내 장치  (3) 차단법: 살정자제와 콘돔 / 살정자제와 폐쇄캡(피임용질격막 또는 경부/구개캡)  (4) 남성의 정관절제술 |
| **적용방법** | 본 연구는 혈관 수축성 협심증이 있는 고혈압 환자에서 네비보롤의 효능 및 안전성을 평가하기 위한 예비(pilot) 시험으로써 환자군을 1:1:1 비율이 되도록 무작위 배정하여 3개 군으로 나누어 진행 및 결과를 비교하고자 한다.  ◇ 네비보롤군(Group 1): 경구용 네비보롤(네비레트정) 5mg/일(2주) → 10mg/일(10주)  ◇ 딜티아젬군(Group 2): 경구용 딜티아젬(헤르벤서방정) 90mg/일(2주) → 180mg/일(10주)  ◇ 네비보롤+딜티아젬군(Group 3): 경구용 네비보롤(네비레트정) 2.5mg/일+경구용 딜티아젬(헤르벤서방정) 45mg/일(2주) → 경구용 네비보롤(네비레트정) 5mg/일+경구용 딜티아젬(헤르벤서방정) 90mg/일(10주)  각 기간별로 임상시험용의약품을 식사와 관계없이 1일 1회 아침에 경구 복용한다. |
| **시험기간** | IRB 승인일로부터 2년간 |
| **평가 항목 및**  **평가 방법** | 1. 일차 유효성 평가변수  (1) 베이스라인 대비 12주째 msSBP와 msDBP 변화량  (2) 베이스라인 대비 12주째 협심증 삶의 질 변화량  2. 이차 유효성 평가변수  (1) 베이스라인 대비 12주째 관동맥 연축 변화량  (2) 베이스라인 대비 6주째 msSBP 및 msDBP변화량  (3) 베이스라인 대비 6주, 12주 시점에서 목표혈압에 도달한 비율  (4) 2주 대비 12주째의 msSBP 및 msDBP 변화량  (목표 혈압*: msSBP/DBP < 140/90mmHg)  3. 안전성 평가 변수  이상반응, 실험실적 검사, 신체검사, 활력징후(맥박), ECG |
| **통계분석** | 1. 일차 평가변수  1) 베이스라인 대비 12주째 msSBP와 msDBP 변화량  msSBP와 msDBP에 대하여 베이스라인과 12주 결과의 변화량에 대한 기술통계량(관측 대상 수, 평균, 표준편차, 중앙값, 최소값, 최대값)을 치료군별로 제시하고, 평균 변화량에 대한 세 군간의 비교는 ANOVA 또는 Kruskal-Wallis test를 이용하여 분석한다. 각 군내 변화는 Paired t-test 또는 Wilcoxon signed rank test를 통해 분석한다. 영향 주는 factor 가 더 있을 때에는 ANCOVA 분석을 시행한다.  2) 베이스라인 대비 12주째 협심증 삶의 질 변화량  베이스라인과 12주째 시애틀 협심증 설문지를 통해 합산된 총 점수의 평균을 비교한다. 평균 변화량에 대한 세 군간의 비교는 ANOVA 또는 Kruskal-Wallis test를 이용하여 분석한다. 각 군내 변화는 Paired t-test 또는 Wilcoxon signed rank test를 통해 분석한다. 영향 주는 factor 가 더 있을 때에는 ANCOVA 분석을 시행한다.  2. 이차 평가변수  1) 베이스라인 대비 12주째 관동맥 연축 변화량  관동맥 연축에 대하여 베이스라인과 12주 결과의 변화량에 대한 기술통계량(관측 대상 수, 평균, 표준편차, 중앙값, 최소값, 최대값)을 치료군별로 제시하고, 평균 변화량에 대한 세 군간의 비교는 ANOVA 또는 Kruskal-Wallis test를 이용하여 분석한다. 각 군내 변화는 Paired t-test 또는 Wilcoxon signed rank test를 통해 분석한다. 영향 주는 factor 가 더 있을 때에는 ANCOVA 분석을 시행한다.  2) 베이스라인 대비 6주째 msSBP 및 msDBP 변화량  msSBP및 msDBP에 대하여 베이스라인과 6주 결과의 변화량에 대한 기술통계량(관측 대상 수, 평균, 표준편차, 중앙값, 최소값, 최대값)을 치료군별로 제시하고, 평균 변화량에 대한 세 군간의 비교는 ANOVA 또는 Kruskal-Wallis test를 이용하여 분석한다. 각 군내 변화는 Paired t-test 또는 Wilcoxon signed rank test를 통해 분석한다. 영향 주는 factor 가 더 있을 때에는 ANCOVA 분석을 시행한다.  3) 베이스라인 대비 6, 12주 시점에서 목표혈압에 도달한 비율  베이스라인 대비 6, 12주 시점에서 목표혈압에 도달한 비율에 대하여 기술통계량(빈도와 백분율)을 치료군별로 제시하고, 세 군간의 동질성에 대해 Pearson’s chi-square test 또는 Fisher’s exact test를 이용하여 분석한다.  4) 2주 대비 12주째의 msSBP 및 msDBP 변화량  msSBP 및 msDBP에 대하여 2주, 12주 결과의 변화량에 대한 기술통계량(관측 대상 수, 평균, 표준편차, 중앙값, 최소값, 최대값)을 치료군별로 제시하고, 평균 변화량에 대한 세 군간의 비교는 ANOVA 또는 Kruskal-Wallis test를 이용하여 분석한다. 각 군내 변화는 Paired t-test 또는 Wilcoxon signed rank test를 통해 분석한다. 영향 주는 factor 가 더 있을 때에는 ANCOVA 분석을 시행한다.  3. 안전성 평가 변수  치료군 별 약물 투여 후 발생한 이상반응, 이상약물반응(ADR), 중대한 이상반응(SAE)에 대하여 치료군별로 기술통계량(발현 대상자수, 발현율 및 발현 건수)과 발현율에 대한 95% 신뢰구간을 제시하고 치료군간 발현율 차이는 Pearson’s chi-square test 또는 Fisher’s exact test를 이용하여 비교 분석한다.  그외 실험실 검사치 및 활력징후 등에 대하여 결과치가 연속형 변수인 경우 약물 투여 전 대비 약물 투여 12주 후 변화량에 대해 치료군별로 기술통계량(관측 대상 수, 평균, 표준편차, 중앙값, 최소값, 최대값)을 제시하고 ANOVA 또는 Kruskal-Wallis test를 이용하여 분석한다. 각 군내 변화는 Paired t-test 또는 Wilcoxon signed rank test를 통해 분석한다. 결과가 범주형 변수인 경우, 약물 투여 전후 변화에 대한 분할표를 제시하고 군내 변화는 McNemar’s test를 이용하여 분석하고, 군간 차이는 Pearson’s chi-square test 또는 Fisher’s exact test를 이용하여 분석한다. |

**[임상시험 진행 일정표]**

| 일정 | Visit 1 | Visit 2^a^ | Visit 3 | Visit 4 | Visit 5 |
| --- | --- | --- | --- | --- | --- |
| Week (Day) | Week -4 ~ | Baseline  (Day 0) | Week 2  (Day 14)+5 | Week 6  (Day 42)+5 | Week 12  (Day 84)+14 |
| 동의서 취득 | ● |  |  |  |  |
| 선정/제외기준 확인 | ● |  |  |  |  |
| 인구통계학적 조사 | ● |  |  |  |  |
| 병력 조사 | ● |  |  |  |  |
| 체중^b^, 혈압 및 심박수 측정 | ● | ● | ● | ● | ● |
| 임신검사 | ●^c^ |  |  |  |  |
| 무작위 배정 |  | ● |  |  |  |
| 임상시험용의약품 처방 |  | ● | ● |  |  |
| 관상동맥조영술 및 혈관수축유발 검사 | ●^d^ |  |  |  | ● |
| 시애틀 협심증 설문지 조사 |  | ● |  |  | ● |
| 선행^e^/병용약물 확인 | ● | ● | ● | ● | ● |
| 이상반응 확인 |  | ● | ● | ● | ● |
| 반납약 회수 및 순응도 평가 |  |  | ● |  | ● |

a. 스크리닝 시 선정/제외 기준 평가가 가능한 경우 Screening과 Baseline 방문 및 절차는 동일한 날에 시행 가능하다.

b. 체중은 스크리닝 및 Visit 5에만 조사한다.

c. 임신검사 결과가 양성인 가임여성은 임상시험에 참여할 수 없다.

d. 연구참여 전 일상 진료상 필요하여 해당 검사가 진행된 환자를 대상으로 모집한다. Screening 이전 4주 이내의 검사치는 활용 가능하다.

e. 선행약물은 스크리닝 시에만 조사한다.

목 차

[**1. 임상시험의 명칭 및 단계** 8](#_Toc424297863)

[**2. 임상시험 실시기관명 및 주소** 8](#_Toc424297864)

[**3. 임상시험 책임연구자, 담당자, 공동연구자의 성명 및 직명** 8](#_Toc424297865)

[**4. 임상시험 의뢰자명 및 주소** 8](#_Toc424297866)

[**5. 임상시험 연구비, 임상시험요으이갸품지원기관명 및 주소** 8](#_Toc424297867)

[**6. 임상시험의 목적 및 배경** 8](#_Toc424297868)

[**7. 임상시험약의 코드명(또는 주성분의 일반명), 원료약품 및 그 분량, 제형 등(해당되는 경우)** 10](#_Toc424297869)

[**8. 임상시험용의약품** 10](#_Toc424297870)

[**9. 시험대상질환** 11](#_Toc424297871)

[**10. 시험대상자의 선정기준, 제외기준** 11](#_Toc424297872)

[**11. 목표한 시험대상자의 수 및 그 근거** 12](#_Toc424297873)

[**12. 임상시험의 기간** 12](#_Toc424297874)

[**13. 임상시험의 방법** 12](#_Toc424297875)

[**14. 관찰항목, 임상검사항목 및 관찰검사방법** 13](#_Toc424297876)

[**15. 예측 이상반응 및 사용상의 주의사항** 16](#_Toc424297877)

[**16. 계획서 위반, 중지 및 탈락 기준** 24](#_Toc424297878)

[**17. 평가변수** 24](#_Toc424297879)

[**18. 평가기준, 평가방법 및 해석방법(통계분석방법)** 24](#_Toc424297880)

[**19. 부작용을 포함한 안전성의 평가기준, 평가방법 및 보고방법** 26](#_Toc424297881)

[**20. 임상시험 후 시험대상자의 진료 및 치료기준** 30](#_Toc424297882)

[**21. 시험대상자의 안전보호에 관한 대책** 30](#_Toc424297883)

[**22. 그 밖에 임상시험을 안전하게 과학적으로 실시하기 위하여 필요한 사항** 31](#_Toc424297884)

[**23. 참고문헌** 32](#_Toc424297885)

시험대상자 설명문 및 동의서: 별첨 1

피해자보상에 대한 규약: 별첨 2

임상시험 참여자: 별첨 3

# **1. 임상시험의 명칭 및 단계**

국문: 혈관 수축성 협심증을 동반한 고혈압 환자에서 네비보롤(Nebivolol)의 효과에 대한 예비(pilot) 시험

영문: A pilot study for the Effect of Nebivolol on Coronary Arterial Spasm in Patients with Hypertension

* 전향적, 무작위배정, 공개, 연구자주도, 예비(pilot) 시험

# **2. 임상시험 실시기관명 및 주소**

1) 고려대학교 구로병원 심혈관센터, 서울 구로구 구로동길 97

2) 고려대학교 안암병원 순환기내과, 서울시 성북구 인촌로 73

3) 연세의료원 신촌세브란스병원, 서울시 서대문구 연세로 50-1

# **3. 임상시험 책임연구자, 담당자, 공동연구자의 성명 및 직명**

3.1 임상시험 책임자

| 고려대학교 구로병원 | 박창규 / 책임연구자 / 순환기내과 교수 |
| --- | --- |
| 고려대학교 안암병원 | 홍순준 / 책임연구자 / 순환기내과 교수 |
| 연세의료원 세브란스병원 | 김중선 / 책임연구자 / 심장내과 교수 |

3.2 임상시험 담당자, 관리약사, 코디네이터

별첨 3. 임상시험 참여자 참고

# **4. 임상시험 의뢰자명 및 주소**

고려대학교 구로병원 심혈관센터 박창규 (순환기내과 교수), 서울 구로구 구로동길 97

# **5. 임상시험 연구비 및 임상시험용의약품 지원기관**

한국메나리니(주), 서울특별시 강남구 테헤란로 411, 성담빌딩 12층

# **6. 임상시험의 목적 및 배경**


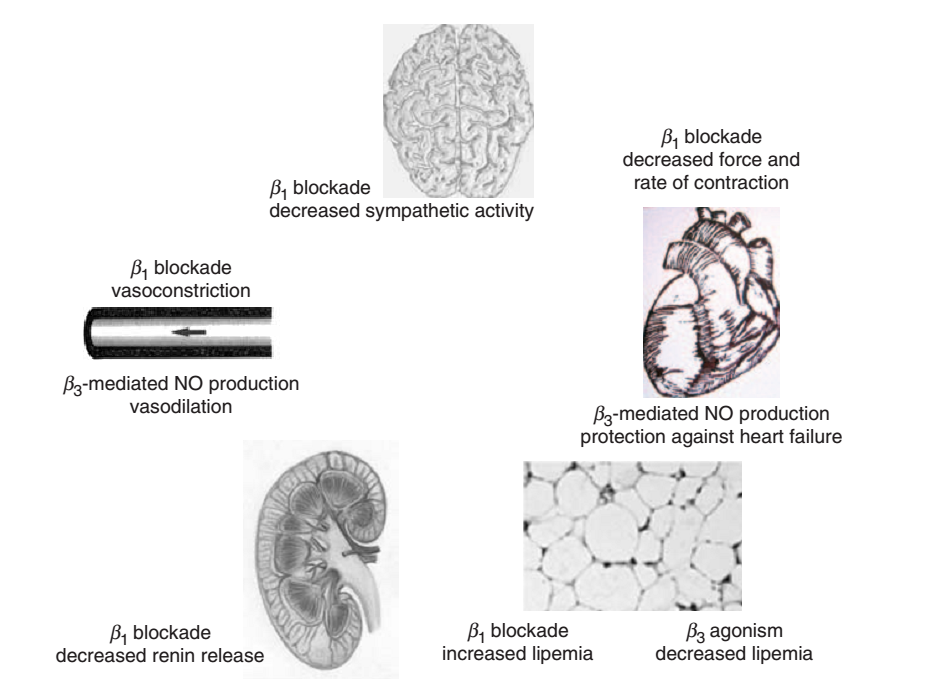
혈관 내막의 기능 장애 (endothelial dysfunction; 혈관 내피 세포에서 기시하는 일산화질소 (Nitric oxide) 기능의 손상)와 관상 동맥 질환의 위험도와의 상관관계는 기존의 연구들을 통해 잘 알려져 있다.^1,2^ 혈관 내막에 작용 하는 일산화질소(NO)의 기능 저하는 주로 합성 저하, 산화 스트레스(Oxidative stress)로 인한 손실, 혈관 확장 작용에 대한 감수성 감소 등으로 설명된다.^3,4^ 특히, 고혈압 환자는 동물 실험 및 여러 임상 연구를 통해 혈관 내막 기능에 장애가 발생하는 것으로 알려졌고, 이는 주로 혈관내의 생체 역학적 마찰력의 증가 및 일산화질소의 생물학적 가용능의 감소에 기인하며, 이는 결국 혈관 내막의 일산화질소 생성의 부조화 및 정상 혈관 확장능의 변화를 유발하게 된다.^5^ 또한, 최근에는 내막 기능 장애에 대한 조기 진단 및 치료가 내막 기능 장애를 호전시킬 수 있고, 관상 동맥 질환의 진행을 예방한다는 보고들이 있다.^6^ 그러나, 이러한 내막 기능의 장애가 있는 혈관 수축성 협심증 환자에서 선택할 수 있는 약제는 매우 제한적인 것이 현실이다. 최근까지 베타 차단제는 아드레날린 자극에 대한 혈관 확장 작용을 억제한다는 보고가 있어 혈관 수축성 협심증 환자에서 상대적 금기에 해당하는 약제였으며, 한 연구에서는 프로프라놀롤(propranolol)이 혈관 수축성 협심증을 억제하지 못할 뿐만 아니라 오히려 악화시킨다는 보고가 있었다.^7^ 하지만, 최근에 개발된 3세대 베타 차단제(예: 카베디롤(carvedilol), 네비보롤(nebivolol))들의 혈관 확장 작용에 대한 보고들이 잇따르면서 혈관 수축성 협심증에서의 베타 차단제의 역할이 재조명되고 있으며, 특히, 네비보롤 (선택적, 지속형 베타차단제)은 β-1 아드레날린 수용체 차단 및 혈관 내막에 작용하여 혈관 확장 작용을 하는 것으로 알려져 있으며, 또한 β-3 아드레날린 수용체를 자극하여 혈관 내막의 일산화 질소 생성 및 항산화 효과를 유발하는 작용도 하는 것으로 알려져 있다.(그림)^8-11^ 따라서, 본 임상 시험은 네비보롤이 고혈압 환자에서 혈관 수축을 억제하고, 혈관 수축성 협심증 환자에서 효과가 있을 것이라는 가정 하에 예비(pilot) 시험을 진행하고자 한다.

그림. 주요 장기에 작용하는 네비보롤(Nebivolol)의 기전 및 효과.^11^ 네비보롤은 β-1 아드레날린 수용체를 선택적으로 차단하면서 혈관 수축을 억제하는 효과를 나타내며, 특히 심장에서 β-3 아드레날린 수용체를 자극하는 효과와 함께 나타나게 되어 효과가 증대된다.

# **7. 임상시험약의 코드명(또는 주성분의 일반명), 원료약품 및 그 분량, 제형 등(해당되는 경우)**

1) 네비레트정 5mg

(1) 제품명: 네비레트정(네비보롤염산염)

(2) 제형 및 성상: 흰색의 원형 정제

(3) 함량: 네비보롤염산염 5.45mg (네비보롤 5mg)

(4) 저장방법: 기밀용기, 실온(1-30℃)보관

2) 헤르벤서방정 90mg

(1) 제품명: 헤르벤서방정(딜티아젬염산염)

(2) 제형 및 성상: 흰색-유백색의 원형서방정

(3) 함량: 딜티아젬염산염 90mg

(4) 저장방법: 기밀용기, 실온보존(1-30℃)

# **8. 임상시험용의약품**

8.1 임상시험용의약품의 라벨

1) "임상시험용"이라는 표시

2) 제품의 코드명 또는 주성분의 일반명

3) 제조번호 및 사용(유효)기한 또는 재검사일자

4) 저장방법

5) 임상시험계획 승인을 받은 자의 상호와 주소

6) "임상시험 외의 목적으로 사용할 수 없음"이라는 표시

8.2 임상시험의약품의 교부, 보관, 관리 및 기록

시험자는 임상시험에 사용되는 의약품의 수령, 운송량 및 상태를 파악하고, 임상시험에 사용되는 의약품 수령, 교부 및 반납기록을 보관해야 한다.

1) 교부

임상시험에 사용되는 의약품의 불출은 본 임상시험에 참여하고 있는 시험책임자 또는 시험담당자의 서명이 있는 처방전에 의해 행해져야 한다.

2) 보관, 관리 및 기록

임상시험용의약품은 각 임상시험실시기관에서 지정된 사람이 수령하여, 안전하고 적절하게 취급 및 보관해야 하며, 지정된 사람만이 접근할 수 있는 안전한 장소에 보관해야 한다. 수령 후 임상시험용의약품 라벨에 명시된 지시사항에 따라 보관해야 한다.

임상시험 관리약사는 의약품 수불대장에 시험대상자의 배정번호, 처방날짜, 불출수량 등을 기록하여 보관, 관리하며 사용 상태를 파악하고 임상시험에 사용되는 의약품 수령, 교부 및 반납 기록 목록을 보관하여야 한다.

시험자는 임상시험용의약품과 이와 관련된 물품을 임상시험계획서 상에 명시된 내용 이외의 용도로 사용해서는 안 된다.

3) 미사용 의약품의 반납

임상시험 관리약사는 지원기관에서 제공한 임상시험용의약품에 한해서 미사용 의약품을 지원기관에 반납한다. 임상시험실시기관에서 임상시험용의약품을 폐기 또는 분실하는 경우 관리자가 그러한 사항을 임상시험용의약품 관리표 등에 기록한다. 임상시험종료 시에는 시험대상자에게 교부되지 않은 임상시험에 사용되는 의약품과 반납약을 포함한 모든 미사용 임상시험용의약품을 지원기관에 반환하여야 한다. 임상시험에 사용되는 의약품 발송 시에는 반환되는 약을 확인할 수 있는 배송기록을 첨부한다.

# **9. 시험대상질환**

혈관 수축성 협심증을 동반한 고혈압 환자

# **10. 시험대상자의 선정기준, 제외기준**

10.1 선정기준

1) 만 20세~80세의 고혈압(stage I-2: 수축기 혈압 140-179mmHg 및 이완기 혈압 90-109mmHg) 환자들 중 관상동맥조영술 및 혈관수축유발 검사를 통해 혈관 수축성 협심증을 진단 받은 환자로 통원치료가 가능한 자

2) 본 임상시험에 참여를 결정하며 자발적으로 동의서에 서명한 자

10.2 제외기준

1) 베타 차단제 혹은 칼슘 차단제에 과민 반응을 보인 기왕력

2) 치매 또는 동반된 정신과 질환이 있거나 약물 오남용의 기왕력

3) 스크리닝 전 1개월 이내에 다른 임상시험에 참여한 자

4) 계획서의 준수사항과 진행 절차를 이행할 수 없거나 참여에 부적합한 의학적 상태로 시험자가 판단한 자

5) 연구약물의 약효평가에 영향을 줄 수 있는 약(ACE 억제제, 안지오텐신차단제, 임상시험용의약품외 베타차단제, 임상시험용의약품외 칼슘길항제, 인다파마이드를 제외한 이뇨제)을 복용하고 있는 시험대상자 (이러한 시험대상자는 최소한 2주간의 wash-out period 후 참여 가능)

6) 악성 고혈압 (망막 출혈 혹은 유두 부종이 있는 경우) 혹은 알려진 중등증 또는 악성 망막증 (최근 6개월 이내 망막 출혈, 시력 장애, 망막 미세동맥류)

7) 이차성 고혈압의 과거력 및 이차성 고혈압이 의심되는 모든 병력: 대동맥축착증, 고알도스테론혈증, 신동맥협착, 쿠싱병, 크롬친화세포종, 다낭성 신질환 등

8) 증상을 동반한 기립성 저혈압 환자

9) 중증의 심장 질환자 (심부전(NYHA class 3와 4), 최근 6개월 이내 허혈성 심장질환 (협심증, 심근경색), 경피적 관상동맥 확장술, 또는 관상동맥 우회술 치료 등을 받은 자)

10) 중증의 뇌혈관장애 환자 (최근 6개월 이내 뇌졸중, 뇌경색, 뇌출혈 등)

11) 무뇨 또는 중증의 신부전 환자 (creatinine clearance < 30mL/min)

12) 중증 간부전이거나 AST 또는 ALT > 정상 상한치의 3배, 담도 폐쇄 담즙성 간경변, 담즙 정체 환자

13) 약물의 흡수, 분포, 대사, 배설에 영향을 줄 수 있는 위장관 질환 및 수술 환자, 시험자가 임상적으로 유의하다고 판단하는 현재의 활동성 위염 및 위장관/직장 출혈, 최근 12개월 이내의 활동성 염증성 대장 증후군 등.

14) 임부 및 수유부, 임상시험 기간 동안 임신 계획이 있거나 적절한 피임방법에 동의하지 않는 가임 여성

* 적절한 피임방법은 다음과 같다.

(1) Progestin 단일제의 호르몬 요법 (경구, 이식정)

(2) 자궁내 장치

(3) 차단법: 살정자제와 콘돔 / 살정자제와 폐쇄캡(피임용질격막 또는 경부/구개캡)

(4) 남성의 정관절제술

# **11. 목표한 시험대상자의 수 및 그 근거**

본 연구는 연구와 관련된 선행 연구가 없으므로 예비 시험(pilot study)에 해당하며, 환자군을 3개의 군으로 나누어 각 군당 36명씩 총 108명의 환자를 모집할 예정이다.

# **12. 임상시험의 기간**

IRB 승인일로부터 2년간

# **13. 임상시험의 방법**

13.1 연구 설계 및 방법

본 연구는 혈관 수축성 협심증이 있는 고혈압 환자에서 네비보롤의 효능 및 안전성을 평가하기 위한 예비(pilot) 시험으로써 환자군을 1:1:1 비율이 되도록 무작위 배정하여 3개 군으로 나누어 진행 및 결과를 비교하고자 한다.

◇ 네비보롤군(Group 1): 경구용 네비보롤(네비레트정) 5mg/일(2주) → 10mg/일(10주)

◇ 딜티아젬군(Group 2): 경구용 딜티아젬(헤르벤서방정) 90mg/일(2주) → 180mg/일(10주)

◇ 네비보롤+딜티아젬군(Group 3): 경구용 네비보롤(네비레트정) 2.5mg/일+경구용 딜티아젬(헤르벤서방정) 45mg/일(2주) → 경구용 네비보롤(네비레트정) 5mg/일+경구용 딜티아젬(헤르벤서방정) 90mg/일(10주)

각 기간별로 임상시험용의약품을 식사와 관계없이 1일 1회 아침에 경구 복용한다.

13.2 무작위 배정 방법

본 임상시험은 선정기준을 만족하고 연구에 참여하기로 동의한 시험대상자의 적합성이 최종적으로 확인되면 시험대상자는 무작위 배정표에 따라 순차적으로 무작위 배정번호를 부여 받는다. 무작위 배정표는 블록 무작위 배정(block randomization) 방법에 의하여 각 군간 비율이 1:1:1로 배정될 수 있도록 하며, 무작위 배정표는 Random allocation software program을 이용하여 프로그램하고, 미리 지정한 블록크기를 고려하여 충분한 크기의 무작위배정번호를 부여한다.

13.3 혈관 수축성 협심증 양성 판단 기준

약물 투여에 따른 관동맥 변화는 동맥 내경을 캘리퍼로 측정하여 computerized quantitative analyzer(QCA)로 분석하였고 조영제로 충만된 카테터의 외경을 캘리퍼로 측정한 값을 참고치로 삼았다. 그 결과 심외막 관동맥에 국소적 혹은 전반적인 70% 이상의 의미있는 변화와 흉통이 있을 때 또는 심전도 상의 허혈성 변화 (ST분절의 상승 혹은 1mm 이상의 하강, T파의 역위)가 있을 때로 정한다. 또한, 혈관 수축율은 [(나이트로 글리세린 투여 후 내경 – 아세틸콜린 투여 후 내경)/나이트로 글리세린 투여 후 내경 X100]으로 정의하였다.

13.4 병용요법 및 금기약물

시험대상자는 시험기간 중 임상시험용 의약품을 제외하고는 시험 결과에 영향을 미칠 수 있는 다른 약물을 투여 받을 수 없다. 단, 투여 6주 이후 SBP 140, DBP 90 이상일 경우 인다파마이드(1.25mg/day po)를 허용할 수 있으며, 필요한 경우 연구자의 판단에 따라 시험 결과에 영향을 미치지 않는 약물로 판단되는 경우 허용할 수 있다. 임상시험 기간 중 허용여부에 상관없이 병용 약물은 약제명, 일일투여량, 투여목적, 사용기간을 증례기록서에 반드시 기록한다.

금기약물:

- ACE 억제제
- 안지오텐신차단제
- 임상시험용의약품외 베타차단제
- 임상시험용의약품외 칼슘길항제
- 인다파마이드를 제외한 이뇨제

# **14. 관찰항목, 임상검사항목 및 관찰검사방법**

14.1 임상시험 진행 일정표

| 일정 | Visit 1 | Visit 2^a^ | Visit 3 | Visit 4 | Visit 5 |
| --- | --- | --- | --- | --- | --- |
| Week (Day) | Week -4 ~ | Baseline  (Day 0) | Week 2  (Day 14)+5 | Week 6  (Day 42)+5 | Week 12  (Day 84)+14 |
| 동의서 취득 | ● |  |  |  |  |
| 선정/제외기준 확인 | ● |  |  |  |  |
| 인구통계학적 조사 | ● |  |  |  |  |
| 병력 조사 | ● |  |  |  |  |
| 체중^b^, 혈압 및 심박수 측정 | ● | ● | ● | ● | ● |
| 임신검사 | ●^c^ |  |  |  |  |
| 무작위 배정 |  | ● |  |  |  |
| 임상시험용의약품 처방 |  | ● | ● |  |  |
| 관상동맥조영술 및 혈관수축유발 검사 | ●^d^ |  |  |  | ● |
| 시애틀 협심증 설문지 조사 |  | ● |  |  | ● |
| 선행^e^/병용약물 확인 | ● | ● | ● | ● | ● |
| 이상반응 확인 |  | ● | ● | ● | ● |
| 반납약 회수 및 순응도 평가 |  |  | ● |  | ● |

a. 스크리닝 시 선정/제외 기준 평가가 가능한 경우 Screening과 Baseline 방문 및 절차는 동일한 날에 시행 가능하다.

b. 체중은 스크리닝 및 Visit 5에만 조사한다.

c. 임신검사 결과가 양성인 가임여성은 임상시험에 참여할 수 없다.

d. 연구참여 전 일상 진료상 필요하여 해당 검사가 진행된 환자를 대상으로 모집한다. Screening 이전 4주 이내의 검사치는 활용 가능하다.

e. 선행약물은 스크리닝 시에만 조사한다.

14.2 관찰항목

14.2.1 서면동의 및 스크리닝 번호 부여

관상동맥조영술 및 혈관수축유발 검사가 진행된 환자를 대상으로 연구자에 의해 연구 참가가 적절하다고 판단되는 환자를 선정하여, 시험대상자 설명문을 통해 본 임상시험 관련 모든 정보를 제공하고 시험대상자의 성명, 서명, 날짜가 포함된 시험대상자 동의서를 득한다. 시험대상자 동의서를 득한 순서대로 스크리닝 번호를 부여한다. 스크리닝 번호는 아래와 같이 부여한다.

- SXXZZZ (S: screening의 첫글자, XX: 기관번호, ZZZ: 일련번호)

시험대상자 동의는 어떠한 임상시험 과정보다 앞서 이루어져야 한다. 동의서는 시험대상자 또는 법적 대리인에 의해 성명, 서명, 날짜가 자필로 기록되어야 하며, 이는 시험대상자의 자발적 동의임을 의미한다. 또한, 본 임상시험의 시험자는 시험대상자 동의를 확인하는 성명, 서명, 날짜를 시험대상자가 작성한 동의서에 함께 기록해야 하며 동의서의 사본 1부를 시험대상자에게 교부하고 원본 1부를 시험자 파일에 보관한다.

14.2.2 등록번호 부여

시험대상자가 본 연구계획서 상 모든 선정기준을 만족하고, 제외기준에 해당되지 않는 경우 시험책임자는 임상시험용의약품을 처방하는 순서대로 등록번호를 부여한다. 이때 등록번호는 치료군 배정순서와 일치한다.

14.2.3 인구통계학적 조사
서면 동의한 시험대상자의 생년월일, 성별, 신장, 체중, 허리 둘레, 엉덩이 둘레, 목 둘레를 조사한다.

14.2.4 병력 조사
스크리닝 번호를 부여받은 시험대상자의 병력에 대해서 조사한다. 흡연, 음주력, 만성질환 가족력, 과거력 및 현재병력의 유무를 조사한다.

14.2.5 진료실 혈압(clinic BP) 및 맥박 측정
혈압 및 맥박은 내원 시 다른 검사 실시 이전에 측정하며, 안정 시 좌위 혈압 및 맥박을 측정한다.

혈압은 최소 5분 이상의 휴식을 취한 후에 앉은 자세로 양팔을 2분 간격으로 2회씩 수축기 및 이완기 혈압을 측정한다. 2회 측정한 이완기 혈압 차가 10mmHg 이상이면 한번 더 측정하여 2, 3번째 혈압의 평균값을 기록한다. 베이스라인 방문 시 양쪽 팔의 상완부에서 혈압을 측정하고 이후부터는 혈압이 높았던 팔의 혈압만을 측정한다.

14.2.6 임신검사

폐경이 확인된 시험대상자 또는 불임수술을 받은 시험대상자를 제외한 가임 연령의 여성에 한하여 소변검사(urine-HCG)를 통해 임신여부를 확인한다.

14.2.7 관상동맥조영술 및 혈관수축유발 검사

본 연구는 연구참여 전 일상적인 진료하에서 관상동맥조영술 및 혈관수축유발 검사를 통해 혈관수축성 협심증을 진단 받은 환자를 대상으로 한다.

일반적인 관상동맥조영술 및 혈관수축유발 검사는 다음과 같이 진행된다.:

대상 환자는 혈관 수축 유발 검사에 영향을 줄 수 있는 약제들 (예: nitrate계, 칼슘 채널 차단제, 베타 차단제 등)을 검사 시행 48시간 전에 중단 시킨다. 관상동맥조영술은 요골 동맥 혹은 대퇴 동맥을 천자한 후 Judkins 카테터를 삽입하여 촬영한다. 관상동맥조영술 전에 유발 검사 중의 심전도 변화를 기록하기 위해 심전도를 부착한다. 조영제는 비이온화 제품인 ultravist를 사용하고 혈관 수축 유발을 위해 일본 동경 제일약품사 제품인 아세틸콜린을 생리식염수에 희석시켜 사용한다. 아세틸콜린 투여 용량은 좌 관상동맥에 10ug(A1), 25ug(A2), 50ug(A3)의 순으로 순차적으로 증량하여 약 60초에 걸쳐서 서서히 주사하고, 매회 아세틸콜린 투여는 2분 간격으로 진행한다. 유발 검사가 종료되거나 아세틸콜린 약물 투여 후 의미있는 관상동맥 수축, 흉통, 심전도 상의 변화가 생기면 nitroglycerin 200ug을 관동맥 내에 주입한다.

14.2.8 시애틀 협심증 설문지 (Seattle Angina Questionnaire)
본 설문지는 허혈성 심장질환 환자의 치료 효과 판정 수단으로 이용하기 위해 개발되었으며 협심증 관련 삶의 질을 구체적으로 평가할 수 있다는 장점이 있다. 총 5가지 범주의 19항목으로 설문이 이루어져 있으며, 각 범주에 대해 0점 (worst) ~ 100 (best) 로 점수를 산출하고 합산하는 방식이다. 본 연구에서는 약물치료 시작 전과 치료 종료 시점인 3개월에 설문 조사를 시행할 예정이다.

14.2.9 선행/병용약물 확인

선행 약물은 스크리닝 전 4주 이내 투여한 약물에 대해 조사한다. 병용약물은 이전 방문일 이후 투여된 병용약물을 조사한다.

14.2.10 이상반응 평가

임상시험용의약품 투여 후 시험대상자에게 나타난 이상반응에 대해 평가하고 기록한다.

본 연구 진행 과정 외 시험대상자의 일상적인 진료 하에서 실험실적 검사, 심전도 검사, 심초음파 검사가 진행된 경우, 연구자는 해당 검사 결과치를 참고하여 임상시험용의약품 투여 전 대비 투여완료 시점의 임상적으로 유의한 결과 변화에 대해 이상반응으로 판단하고 평가할 수 있다.

# **15. 예측 이상반응 및 사용상의 주의사항**

본 임상시험에 사용되는 임상시험용의약품은 모두 시판 허가된 의약품으로 각 사용상의 주의사항은 다음과 같다.

15.1 네비레트정 사용상의 주의사항

1. 다음 환자에게는 투여하지 말 것.

1) 이 약에 과민증 환자

2) 기관지경련 또는 기관지천식의 병력을 가진 환자

3) 대사성 산증 환자

4) 서맥(< 60 bpm), 2~3도의 방실차단환자

5) 급성 심부전, 심인성 쇽 또는 inotropic 치료(정맥주사)를 필요로 하는 대상부전성 심부전 환자

6) 조절되지 않는 심부전환자

7) 저혈압 환자 (수축기혈압 < 90mmHg)

8) 중증의 말초순환장애 환자

9) 동방차단을 포함한 동기능부전증후군 환자

10) 치료되지 않는 크롬친화 세포종환자

11) 간부전 또는 간기능 손상 환자

12) 임산부 또는 수유부

13) 중증의 신부전 (혈청 크레아티닌 ≥2.5 mg/dL)을 가진 만성 신부전 환자

14) 이 약은 유당을 함유하고 있으므로, 갈락토오스 불내성((galactose intolerance), Lapp 유당분해효소 결핍증(Lapp lactase deficiency) 또는 포도당-갈락토오스 흡수장애(glucose-galactose malabsorption)등의 유전적인 문제가 있는 환자에게는 투여하면 안 된다.

2. 다음 환자에게는 신중히 투여할 것.

1) 만성 장애성 폐장해 환자

2) 치료되지 않은 울혈성 심부전(CHF) 환자

3) 허혈성 심부전 환자

4) 말초순환장애가 있는 환자 (레이노증후군, 간헐성 파행)

5) 1도의 방실차단 환자

6) 무저항성 α-수용체 매개의 관상동맥 혈관수축에 기인한 Prinzmetal 협심증환자

7) 갑상선 기능항진증 환자 (빈맥 증상을 은폐시킬 수 있다.)

8) 건선의 병력이 있는 환자

9) 당뇨병 환자 (혈당치에는 영향을 미치지 않으나 저혈당의 특정한 증상(빈맥, 두근거림)을 은폐시킬 수 있다.)

10) 고령자

3. 이상반응

이상반응은 고혈압과 만성심부전으로 분리하였다.

<고혈압>

1) 과민증 : 발진이 나타날 수 있다.

2) 눈: 때때로 시력손상이 나타날 수 있다.

3) 순환기계: 때때로 서맥, AV차단, AV전도지연, 저혈압, 심부전, 간헐성 파행(의 증가)이 나타날 수 있다.

4) 정신신경계 : 자주 두통, 어지러움, 감각이상, 때때로 우울, 악몽, 매우 드물게 실신이 나타날 수 있다.

5) 소화기계 : 자주 설사, 변비, 구역, 때때로 소화불량, 고창, 구토가 나타날 수 있다.

6) 호흡기계 : 자주 호흡곤란, 때때로 기관지경련이 나타날 수 있다.

7) 피부 및 피하조직 : 때때로 가려움증, 홍반성 발진, 매우 드물게 건선의 악화가 나타날 수 있다.

8) 기타: 자주 부종, 피로감, 때때로 발기부전이 나타날 수 있다.

9) 면역계 : 빈도불명의 혈관신경성 부종, 과민증이 나타날 수 있다.

10) 다른 베타차단제에서 다음과 같은 이상반응이 보고되어 있다.

① 정신신경계 : 환각, 정신병, 혼미, 청색성 사지

② 눈 : 안구건조, practolol형 안점막피부 독성

③ 기타 : 레이노이드 현상

<만성심부전>

1) 매우 자주 서맥, 어지러움, 자주 심부전의 악화, 기립성저혈압, 약물 불내성, 1도 방실차단, 하지부종이 나타날 수 있다.

<국내 시판후 조사 결과>

국내에서 4년 동안 757명을 대상으로 실시한 사용성적조사 결과 인과관계에 상관없이 유해사례발현율은 25.6%(194/757명, 346건)로 보고되었다.

- 이 약과 인과관계를 배제할 수 없는 약물유해반응 발현율은 15.2%(115/757명, 166건)이었다. 보고된 약물유해반응으로는 어지러움이 2.8% (21명, 21건), 두통이 1.2% (9명, 9건), 변비, 부종이 각각 0.8% (6명, 6건), 구역, 가슴쓰림, 설사, 불면증, 두근거림이 각각 0.7% (5명, 5건), 소화불량, 복통, 서맥이 각각 0.5% (4명, 4건), 구토, 빈뇨, 혈당상승이 각각 0.4% (3명, 3건), 목/어깨통증이 0.3% (2명, 3건), 감각이상, 위염, 가슴통증, 피로감, 무력증, 효과부족, 체중감소, 호흡곤란, 기침, 감기, 건성기침, 식욕부진, 불안, 등통증, 혈중크레아티닌증가, 귀울림이 각각 0.3% (2명, 2건), 현기증, 떨림, 신경근육병증, 위식도역류, 과민성대장증후군, 구갈, 위궤양, 위장장애, 전신쇠약, 얼굴부종, 전신부종, 상기도감염, 비염, 인후통, 콧물, 초조, 우울증, 졸림, 관절통, 옆구리통증, 목경직, 허리통증, BUN증가, 방광불편, 방광염, 요로결석, 혈뇨, 당뇨병, 두드러기, 무좀, 발한, 백선증, 저혈압, 고혈압, 뇌혈관질환, 말초혈관질환, 홍조, 코피, ALT증가, AST증가, 바이러스감염, 갑상선염, 만성골수성백혈병이 각각 0.1% (1명, 1건)순으로 나타났다.

이 중 예상하지 못한 약물유해반응은 가슴쓰림, 불면증, 두근거림이 각각 0.7% (5명, 5건), 복통이 0.5% (4명, 4건), 빈뇨, 혈당상승이 각각 0.4% (3명, 3건), 목/어깨통증이 0.3% (2명, 3건), 위염, 가슴통증, 무력증, 효과부족, 체중감소, 기침, 감기, 건성기침, 식욕부진, 불안, 등통증, 혈중크레아티닌증가, 귀울림이 각각 0.3% (2명, 2건), 현기증, 떨림, 신경근육병증, 위식도역류, 과민성대장증후군, 구갈, 위궤양, 위장장애, 전신쇠약, 전신부종, 상기도감염, 비염, 인후통, 콧물, 초조, 졸림, 관절통, 옆구리통증, 목경직, 허리통증, BUN증가, 방광불편, 방광염, 요로결석, 혈뇨, 당뇨병, 두드러기, 무좀, 발한, 백선증, 고혈압, 뇌혈관질환, 말초혈관질환, 홍조, 코피, ALT증가, AST증가, 바이러스감염, 갑상선염, 만성골수성백혈병이 각각 0.1% (1명, 1건)로 보고되었다.

- 중대한 약물유해반응은 감각이상, 가슴통증, 호흡곤란, 서맥, 뇌혈관질환, 만성골수성백혈병이 각각 1명에서 1건 보고되었고, 이 중 가슴통증, 뇌혈관질환, 만성골수성백혈병은 중대하고 예상하지 못한 약물유해반응이었다.

4. 일반적 주의

다음은 베타차단제에 일반적으로 적용되는 것이다.

1) 베타차단제의 연속적 사용은 마취유도 및 삽관시의 부정맥 위험성이 감소된다. 베타차단제의 사용이 수술준비에 방해될 경우 최소한 24시간 전에 베타차단제를 미리중지해야 한다. 심근 억제를 일으키는 마취제는 주의관찰한다.

미주신경 반응은 아트로핀을 정맥투여하여 억제할 수 있다.

2) 베타차단제는 서맥을 일으킬 수 있으므로 휴식시의 맥박이 50-55bpm으로 떨어지거나 서맥을 일으킬 것으로 판단될 경우에는 용량을 감소시킨다.

3) 베타차단제는 항원에 대한 감수성과 아나필락스 반응의 정도를 증가시킬 수 있다.

4) 허혈성 심질환 환자의 경우, 1~2주 이상의 간격을 두고 베타차단제의 치료를 차츰차츰 중단해야 한다. 협심증의 악화를 예방하기 위해서는 다른 대체치료를 동시에 시작해야 한다

5) 초회투여 및 용량 증량시마다 약물 투여 후 적어도 2시간동안 경험 많은 의사의 감독하에 환자의 임상상태 (특히 혈압, 심박수, 전도 장애, 신기능 악화의 징후)등이 안정적인지 관찰되어야 한다.

6) 약물을 증량하는 동안 심기능의 악화나 불내성이 관찰되면 초회용량을 투여하거나 용량을 감소하여 투여하도록 하며, 필요한 경우 (중증의 저혈압, 급성 폐부종을 동반한 심부전의 악화, 심인성 쇼크, 증상성 서맥 또는 방실 차단의 발생) 즉각적으로 약물투여를 중단한다.

5. 상호작용

약력학적 상호작용 (일반적인 베타차단제의 상호작용)

: 투여가 권장되지 않는 약물

1) 칼슘길항제 (염산베라파밀, 딜티아젬계)와 병용투여시 수축성과 방실 전도에 대한 부정적인 영향이 있을수 있으므로 주의하여야 한다. 베타차단제로 치료를 받고 있는 환자에게 베라파밀을 정맥으로 투여하는 경우 심각한 저혈압과 방실 차단이 일어날 수 있으므로, 이 약 투여환자에게는 베라파밀을 정맥투여하지 않는다.

2) Class 1 항부정맥용약 (퀴니딘, 하이드로퀴니딘, 시벤졸린, 플레카이나이드, 디소피라미드, 리도카인, 멕실레틴, 프로파페논)과 병용투여시 방실전도시간에 대한 영향과 부정적인 변력작용이 증가될 수 있으므로 병용투여를 권장하지않는다.

3) 중추작용 항고혈압약 (클로니딘, 구안파신, 목소니딘, 메칠도파, 릴메니딘)과의 병용투여시 심박동 및 심박출량의 감소, 혈관이완과 같은 중추 교감신경 긴장의 감소에 의한 심부전의 악화가 발생할 수 있다. 이 계열 약물의 갑작스런 투약중단 (특히 베타 차단제의 투약중지 전)에 의해 반동성 고혈압의 위험이 증가할 수 있다. 두 약물의 병용투여는 권장되지 않는다.

: 투여에 신중한 주의를 요하는 약물

4) Class III 항부정맥용약 (아미오다론)과의 병용투여에 의해 방실전도시간에 대한 영향이 증강될 수 있다.

5) 마취제와의 병용사용은 반사성 빈맥을 줄이며 저혈압의 위험성을 증가시킬 수 있으므로 마취의사에게 투여받고 있음을 알려주어야 한다.

6) 인슐린이나 경구용 당뇨병용약과 병용시 혈당치에는 영향을 주지 않지만 저혈당의 특정한 증상(두근거림, 빈맥)을 은폐시킬 수 있다.

7) 항경련제 (바클로펜) 또는 항종양제 (아미포스틴)와 항고혈압약과의 병용투여시 혈압저하가 증대될 수 있으므로, 항고혈압약의 용량 조절이 필요할 수 있다.

: 투여에 주의를 요하는 약물

8) 디기탈리스제제를 베타-차단제와 병용하면 방실전도시간을 연장시킬 수 있다. 그러나 이 약의 임상시험에서 임상적인 상호작용은 보이지 않았고, 디곡신의 약력학에도 영향을 미치지 않았다.

9) 디하이드로피리딘계 칼슘차단제 (암로디핀, 펠로디핀, 라시디핀, 니카르디핀, 니모디핀, 니트렌디핀)과 병용투여시 저혈압의 위험성을 증가시킬수 있다. 또한 심부전환자에서 심실 펌프기능 악화의 위험성도 배제될 수 없다.

10) 항정신병약 및 항우울약 (삼환계, 바르비탈계, 페노치아진계)의 병용투여는 베타차단제의 저혈압 효과를 증강시킬 수 있다.

11) NSAIDs와의 병용사용은 이 약의 혈압강하효과에 영향을 주지 않는다.

12) 교감신경흥분제는 베타 차단제의 효과를 방해할 수 있다.

약동학적 상호작용

13) 이 약은 CYP2D6 동종효소를 통해 대사되므로 주로 이 경로를 통해 대사되는 파록세틴, 플루옥세틴, 치오리다진, 퀴니딘과의 병용투여에 의해 이 약의 혈중농도가 증가되어 과도한 서맥 및 기타 이상반응 발생의 위험성을 증가시킬 수 있다.

14) 시메티딘과의 병용투여시 이약의 혈중농도는 상승하지만 임상효과에는 변함이 없다. 라니티딘과의 병용투여는 이 약의 약동학에 영향을 주지 않는다. 이 약은 음식과 함께 복용하는 경우나 식사간에 제산제를 복용할 때 같이 투여될 수 있다.

15) 니카르디핀과 병용투여시 두 약물의 혈중농도를 약간 증가시키나 임상효과에는 변함이 없다.

16) 알코올, 퓨로세미드 또는 히드로클로로치아짓과의 병용투여는 이 약의 약동학에 영향을 미치지 않는다.

17) 이 약은 와파린의 약동학이나 약력학에 영향을 미치지 않는다.

6. 임부 및 수유부에 대한 투여

1) 임신중의 투여에 대한 안전성은 확립되어 있지 않았다. 베타차단제는 태반환류를 감소시키는데 이것은 자궁내의 태아사망과 미숙 및 조숙 분만을 초래할 수 있다. 또한 부작용(저혈당, 서맥)이 태아와 신생아에서 나타날 수 있다. 출생후 기간에서 신생아의 심장 및 폐합병증의 위험성이 증가된다. 따라서 이 약은 임부 또는 임신하고 있을 가능성이 있는 부인에게 투여하지 않는다.

2) 대부분의 베타차단제 특히 네비보롤과 그 활성대사물과 같은 지방친화성화합물은 유즙을 통과한다. 이 약이 인체 모유로 이행되는지는 알려져 있지 않으나, 동물시험에서 모유중으로의 이행이 보고되어 있으므로 수유중에는 투여하지 않는다.

7. 소아에 대한투여

18세 이하의 소아에 대한 안전성 및 유효성이 확립되어 있지 않으므로, 소아에게 투여하지 않는 것이 권장된다.

8. 고령자에 대한 투여

고령자에는 다음 사항에 주의하여 저용량에서 투여를 시작하는 등 환자의 상태를 관찰하면서 신중히 투여한다.

1) 일반적으로 고령자에서의 과도한 혈압강하는 바람직하지 않다 (뇌경색등이 나타날 수 있다.).

2) 휴약이 필요할 경우에는 천천히 감량한다.

9. 과량투여시의 처치

1) 증상 : 과량투여시 서맥, 저혈압, 기관지 경련, 급성 심부전이 나타날 수 있다.

2) 처치

① 과량투여나 과민성의 경우 환자를 철저히 보호 감독하고 혈당치를 점검한다.

② 위장관에 남아있는 잔여약물 흡수는 위세척, 활성탄, 완화제의 투여로 치료할 수 있다.

③ 서맥이나 광범위한 미주신경반응은 아트로핀이나 메칠아트로핀의 투여로 치료할 수 있다.

④ 저혈압이나 쇼크는 혈장 또는 혈장대용제로 치료하며 필요시 카테콜아민을 사용할 수도 있다.

⑤ β차단효과는 필요효과가 얻어질 때까지 염산 이소프레날린을 약 5ug/분 또는 도부타민을 2.5ug/분의 초기용량을 서서히 정맥투여하여 막을 수 있다. 난치성인 경우에는 이소프레날린을 도파민과 함께 투여할 수 있다. 만약 이것으로도 효과적이지 않을 경우 글루카곤 50-100 ug/kg정맥투여를 고려한다. 필요한 경우 주사는 한시간내에서 반복하며, 필요시 글루카곤 70 ug/kg/h의 정맥주입을 추가할 수 있다. 심한 저항성 서맥을 치료할 경우 페이스메이커를 삽입할 수도 있다. 끝.

15.2 헤르벤 서방정 사용상의 주의사항

1. 다음 환자에는 투여하지 말 것.

1) 중증의 울혈성 심부전 환자(심부전 증상을 악화시킬 수 있다.)

2) 동기능부전증후군, 동방블록, 방실블록(2, 3도) 환자(인공심실박동기를 착용중인 환자는 제외)

3) 저혈압(수축기압 90mmHg 미만) 또는 쇽 환자

4) 이 약에 과민증의 병력이 있는 환자

5) X선 소견상 급성 심근경색 환자 및 폐울혈 환자

6) 임부 또는 임신하고 있을 가능성이 있는 부인

2. 다음 환자에는 신중히 투여할 것.

1) 울혈성 심부전 환자(심부전 증상을 악화시킬 수 있다.)

2) 중증의 간·신부전 환자(약물대사, 배설이 지연되어 작용이 증강될 수 있다.)

3) 방실블록(1도) 환자

3. 부작용

1) 이 약의 1일 용량을 540㎎까지 투여한 결과 가장 흔한 부작용은 비염, 두통, 인후염, 변비, 기침증가, 인플루엔자 증상, 말초성 부종, 근통, 설사, 구토, 부비강염, 무력감, 요통, 구역, 소화불량, 혈관확장, 사고에 의한 상해, 복통, 관절, 불면, 무호흡, 피진, 이명 등이었다.

2) 순환기계 : 방실블록(1도), 부정맥, 서맥, 기립성 저혈압, 빈맥, 안면창백·홍조, 동정지, 동방블록, 심계항진, 심전도이상, ST상승, 흉통, 부종, 울혈성 심부전, 드물게 완전방실블록, 현저한 서맥 등이 나타날 수 있으므로 이상이 인정되는 경우에는 투여를 중지하고 황산아트로핀, 이소프로테레놀 등의 투여와 함께 필요에 따라 심장박동 등의 적절한 처치를 한다.

3) 정신신경계 : 권태감, 두중감, 종아리경련, 무력감, 긴장항진, 감각이상, 어지러움, 혼몽, 두통이 나타날 수 있다.

4) 소화기계 : 구갈, 식욕부진, 치아이상, 트림, 위부불쾌감, 가슴쓰림이 나타날 수 있다.

5) 피부 : 발한, 피부비대, 피부점막안증후군(Stevens-Johnson 증후군), 중독성 표피괴사증(Lyell 증후군)이 나타날 수 있다.

6) 호흡기계 : 비출혈, 기관지염, 호흡장애가 나타날 수 있다.

7) 비뇨생식기계 : 방광염, 신결석, 발기부전, 무월경, 질염, 전립선 질환이 나타날 수 있다.

8) 대사 및 영양장애 : 통풍, 부종이 나타날 수 있다.

9) 근골격계 : 관절통, 점액낭염, 골격통이 나타날 수 있다.

10) 혈액 및 림프계 : 림프선종이 나타날 수 있다.

11) 전신증상 : 동통, 인과성이 결여된 반응, 경통, 목경직, 발열이 나타날 수 있다.

12) 감각기관 : 약시, 귀아픔이 나타날 수 있다.

13) 과민증 : 때때로 발진, 가려움, 또한 드물게 광과민증, 다형성 홍반양 피진, 두드러기 등이 나타날 수 있으므로 이러한 경우에는 투여를 중지한다.

14) 간장 : 드물게 황달, 간종대가 나타날 수 있으므로 이러한 경우에는 투여를 중지한다. 또한 때때로 ALT, AST의 상승이 나타날 수 있다.

15) 기타 : 여성형 유방, 파킨슨증후군, 혈소판감소, 백혈구감소가 나타날 수 있다. 또한 연용에 따라 드물게 치은비후가 나타날 수 있으므로 이러한 경우에는 투여를 중지한다.

4. 일반적 주의

1) 칼슘길항제의 투여를 갑자기 중지할 경우 증상이 악화될 수 있으므로 휴약을 요하는 경우에는 천천히 감량하면서 관찰을 충분히 한다.

2) 심전도 : 이 약은 동기능부전증후군 환자를 제외하고는 동방결절 회복시간을 크게 지연시키지 않으면서 방실결절 복귀기는 연장시킨다. 이 효과는 드물게 비정상적인 서맥(특히 동기능부전증후군 환자), 때때로 방실블록(2, 3도)을 유발할 수 있다.

3) 울혈성 심부전 : 이 약은 동물의 적출조직에서 음성 근변력 작용을 나타내었음에도 불구하고 정상적인 심실기능을 가진 인체의 혈액동력연구에서는 심기능의 감소나 수축력(dP/dT)에 대한 음성작용을 나타내지 않았다. 좌심실기능부전(박출계수 24±6%) 환자에 대한 경구투여의 급성연구는 수축기능의 특별한 감소없이 심실기능계수 증가를 보여주었다. 이미 심실기능 손상이 있었던 환자에게서 울혈성 심부전증의 악화가 보고되었다. 심실기능부전 환자에게 딜티아젬과 β-차단제를 병용투여하는 것은 제한되어 있으므로 병용투여시 주의한다.

4) 저혈압 : 이 약의 투여로 인한 혈압감소는 때때로 증후성 저혈압을 유발할 수 있다.

5) 이 약은 대부분이 간에서 대사되고 신장과 담즙을 통해 배설되므로 지속적으로 다른 약물과 병용투여하는 경우에는 규칙적인 관찰이 필요하다. 특히 간·신기능부전 환자에 투여하는 경우에는 주의한다. 아급성·만성 독성시험(개, 랫트)에서 고용량 투여시 간손상이 나타났다.

6) 이 약은 지속성 방출형 매트릭스로 이미 협착이 있는 환자에서 이 약 투여에 의한 폐색증상이 보고된 바는 없으나 중증의 위장관 협착이 있는 환자에 투여하는 경우에는 주의한다.

7) 혈압강하 효과에 의해 현기증 등이 나타날 수 있으므로 높은 곳에서의 작업, 자동차 운전 등 위험을 동반하는 기계조작시에는 주의해야 한다.

5. 상호작용

1) 다른 항부정맥약(인산디소피라미드)과 테르페나딘의 병용투여시 QT 연장, 심실성 부정맥을 일으켰다는 보고가 있으므로 이 약과 테르페나딘을 병용투여하지 않는다. 또한, 이 약과 아스테미졸의 병용투여시 QT 연장, 심실성 부정맥을 일으킬 수 있으므로 병용투여하지 않는다.

2) 단트롤렌(주입액) : 심실세동이 나타날 수 있으므로 병용투여하지 않는다.

3) 혈압강하제, 질산염제제 : 혈압강하 효과를 증강시킬 수 있으므로 신중히 투여한다.

4) β-차단제, 라우울피아제제, 부정맥용제(아미오다론 등) : 서맥이 나타날 수 있으므로 신중히 투여한다.

5) 디기탈리스제제(디곡신, 메칠디곡신) : 디기탈리스제제의 혈중농도를 상승시킬 수 있으므로 신중히 투여한다.

6) 아프린딘 : 두 약물의 혈중농도를 서로 상승시킬 수 있으므로 신중히 투여한다.

7) 시클로스포린 : 시클로스포린의 혈중농도를 상승시킬 수 있으므로 신중히 투여한다.

8) 리팜피신 : 이 약의 작용을 저하시킬 수 있으므로 신중히 투여한다.

9) 미다졸람, 페니토인 : 미다졸람, 페니토인의 혈중농도를 상승시킬 수 있으므로 신중히 투여한다.

10) 시메티딘 : 이 약의 혈중농도를 상승시킬 수 있으므로 신중히 투여하다.

11) 테오필린 : 테오필린의 대사, 배설이 지연될 수 있으므로 신중히 투여하다.

12) 마취제 : 심자극생성 억제작용, 심전도 억제작용이 증강될 수 있으므로 신중히 투여한다.

13) 디히드로피리딘계 칼슘차단제(니페디핀 등) : 디히드로피리딘계 칼슘차단제의 혈중농도를 상승시킬 수 있으므로 신중히 투여한다.

14) 카르바마제핀 : 카르바마제핀의 혈중농도를 상승시켜 중독증상(졸음, 구역, 구토, 어지러움 등)이 나타날 수 있으므로 신중히 투여한다.

15) 타크롤리무스 : 타크롤리무스의 혈중농도를 상승시킬 수 있으므로 신중히 투여한다.

16) 트리아졸람 : 트리아졸람의 혈중농도를 상승시킬 수 있으므로 신중히 투여한다.

6. 임부 및 수유부에 대한 투여

1) 동물실험(마우스, 랫트, 토끼)에서 골격, 심장, 망막 및 혀에 기형을 나타내는 경향을 보였으며 또한 출생자에 있어서 체중감소 및 생존수의 감소, 분만지연, 사산수의 증가도 보고되어 있으므로 임부 또는 임신하고 있을 가능성이 있는 부인에는 투여하지 않는다.

2) 이 약의 모유중으로의 이행이 보고되어 있으므로 수유중에는 투여를 피하고 부득이한 경우에는 수유를 중단한다.

7. 소아에 대한 투여

소아에 대한 안전성 및 유효성은 확립되어 있지 않다.

8. 과량투여시의 처치

과량투여시에는 초기 처치로 토근을 투여하여 구토를 유발시키나 약물의 흡수를 감소시키기 위해 활성탄을 투여한다. 위세척과 함께 다음과 같은 방법들도 고려할 수 있다.

1) 서맥 : 아트로핀(0.6-1.0㎎)을 투여한다. 만약 미주신경이 차단되는 반응이 없으면 이소프로테레놀을 주의깊게 투여한다.

2) 고도의 방실블록 : 위의 서맥과 같은 방법으로 치료한다. 고도의 방실블록이 고정적인 경우에는 심박동(pacing)과 함께 치료해야 한다.

3) 심부전 : 근변력성 약물(도파민 또는 도부타민)과 이뇨제를 병용투여한다. 강심제, 혈압상승제, 수액 등의 투여와 보조순환을 적용할 수 있다.

4) 저혈압 : 혈관수축제(도파민 또는 주석산노르에피네프린)를 투여한다. 강심제, 혈압상승제, 수액 등의 투여와 보조순환을 적용할 수 있다. 실제 치료와 용량은 담당의사의 판단, 경험 및 임상적인 상태의 정도에 따라 결정한다. 이 약은 광범위한 대사로 인해 상용량 투여 후에 10배 이상의 혈중농도 변화가 관찰되므로 과량투여 진단시 신중을 기한다. 활성탄의 혈액 환류는 약물배설을 촉진시키기 위한 보조치료요법으로 사용되고 있다.

5) 이 약을 경구로 10.8g까지 과량 투여한 경우에 활성탄을 이용한 적절한 처치로 성공적으로 치료한 예가 보고되어 있다.

9. 적용상의 주의(캅셀제에 한함.)

이 약 투여시에는 캅셀을 개봉하거나 씹지 않도록 주의한다.

# **16. 계획서 위반, 중지 및 탈락 기준**

16.1 계획서 위반

임상시험을 진행하는 동안 계획서 위반이 발생된 경우, 시험자는 가능한 빨리 의뢰자에게 위반사실을 알려야하며, 해당 시험대상자가 임상시험을 지속해야 할지, 중단해야 할지를 결정해야 한다. 계획서 위반으로 중도 탈락한 경우 증례기록서에 그 내용을 기록해야 한다.

16.2 중지 및 탈락 기준

- 선정/제외기준에 적합하지 않은 시험대상자가 참여한 경우 및 선정/제외기준을 만족하였으나 이후 계획서 위반이 확인된 경우
- 시험대상자가 임상시험 참여 동의를 철회한 경우
- 시험대상자의 추적이 안되는 경우
- 시험대상자에게 심각한 약물 관련 이상 반응, 부작용이 발생한 경우
- 기타 이유에 따라 필요한 검사 또는 임상시험 진행이 불가능하다고 판단된 경우

# **17. 평가변수**

17.1 일차 유효성 평가변수

(1) 베이스라인 대비 12주째 msSBP와 msDBP 변화량

(2) 베이스라인 대비 12주째 협심증 삶의 질 변화량

17.2 이차 유효성 평가변수

(1) 베이스라인 대비 12주째 관동맥 연축 변화량

12주에 혈관연축 정도를 평가하기 위해 관상동맥 조영술을 한 번 더 시행하는 것은 일반적인 vasospastic angina 환자에서 많이 이루어지지는 않으나 환자가 동의할 경우 혈관연축의 개선 정도를 평가하는 것은 환자의 약물 조절에 도움을 줄 수 있으며, 검사와 관련한 추가적인 비용 부담없이 시행됩니다.

(2) 베이스라인 대비 6주째 msSBP 및 msDBP변화량

(3) 베이스라인 대비 6주, 12주 시점에서 목표혈압에 도달한 비율

(4) 2주 대비 12주째의 msSBP 및 msDBP 변화량

(목표 혈압*: msSBP/DBP < 140/90mmHg)

17.3 안전성 평가 변수

이상반응, 실험실적 검사, 신체검사, 활력징후(맥박), ECG

# **18. 평가기준, 평가방법 및 해석방법(통계분석방법)**

18.1 통계 분석 일반적 원칙

본 임상시험의 대상자로부터 얻어진 자료는 크게 FAS(Full Analysis Set), PP(Per Protocol)으로 나뉜다. 본 임상의 주 분석(main analysis)은 FAS으로 하고 추가분석은 PP로 분석한다. 두 개의 분석 결과를 비교하여 결과가 상이할 때는 각 분석법의 결과를 제시하고 그 이유를 상세히 기술한다. 모든 통계검정은 유의수준 5%에서 양측검정을 원칙으로 한다.

18.2 분석군의 정의

18.2.1 FAS (Full Analysis Set)

무작위배정 후, 최소한 한번이라도 임상시험용의약품을 적용 받았고, 적용 이후 적어도 한 번의 유효성 평가변수가 측정된 시험대상자를 분석에 포함한다.

18.2.2 PP (Per Protocol)

PP는 본 임상시험에서 FAS 분석에 포함되는 시험대상자 중 임상시험 계획서에 따라 임상시험을 완료한 집단을 의미하고 다음의 경우에 해당하는 시험대상자를 제외한다.

1) 연구계획서에 명시한 기간을 채우지 못하고 임상시험에서 중도 탈락한 시험대상자

2) 병용금지약물 투약 및 병용금지 요법을 받은 시험대상자

3) 선정/제외 기준을 위반한 시험대상

4) 그 외 중대한 계획서 위반으로 간주할 수 있는 경우

18.3 결측치 보정 방법

유효성 평가변수에 대해 어떤 시점에서 결측치가 발생되거나 임상시험이 종료되기 전에 시험대상자가 탈락하여 결측이 발생하면 결측값을 대체하지 않고 있는 자료(available data set)를 그대로 사용하여 분석한다.

18.4 중간분석

계획 없음.

18.5 일차 평가변수

18.5.1 베이스라인 대비 12주째 msSBP와 msDBP 변화량

msSBP와 msDBP에 대하여 베이스라인과 12주 결과의 변화량에 대한 기술통계량(관측 대상 수, 평균, 표준편차, 중앙값, 최소값, 최대값)을 치료군별로 제시하고, 평균 변화량에 대한 세 군간의 비교는 ANOVA 또는 Kruskal-Wallis test를 이용하여 분석한다. 각 군내 변화는 Paired t-test 또는 Wilcoxon signed rank test를 통해 분석한다. 영향 주는 factor 가 더 있을 때에는 ANCOVA 분석을 시행한다.

18.5.2 베이스라인 대비 12주째 협심증 삶의 질 변화량

베이스라인과 12주째 시애틀 협심증 설문지를 통해 합산된 총 점수의 평균을 비교한다. 평균 변화량에 대한 세 군간의 비교는 ANOVA 또는 Kruskal-Wallis test를 이용하여 분석한다. 각 군내 변화는 Paired t-test 또는 Wilcoxon signed rank test를 통해 분석한다. 영향 주는 factor 가 더 있을 때에는 ANCOVA 분석을 시행한다.

18.6 이차 평가변수

18.6.1 베이스라인 대비 12주째 관동맥 연축 변화량

관동맥 연축에 대하여 베이스라인과 12주 결과의 변화량에 대한 기술통계량(관측 대상 수, 평균, 표준편차, 중앙값, 최소값, 최대값)을 치료군별로 제시하고, 평균 변화량에 대한 세 군간의 비교는 ANOVA 또는 Kruskal-Wallis test를 이용하여 분석한다. 각 군내 변화는 Paired t-test 또는 Wilcoxon signed rank test를 통해 분석한다. 영향 주는 factor 가 더 있을 때에는 ANCOVA 분석을 시행한다.

18.6.2 베이스라인 대비 6주째 msSBP 및 msDBP 변화량

msSBP및 msDBP에 대하여 베이스라인과 6주 결과의 변화량에 대한 기술통계량(관측 대상 수, 평균, 표준편차, 중앙값, 최소값, 최대값)을 치료군별로 제시하고, 평균 변화량에 대한 세 군간의 비교는 ANOVA 또는 Kruskal-Wallis test를 이용하여 분석한다. 각 군내 변화는 Paired t-test 또는 Wilcoxon signed rank test를 통해 분석한다. 영향 주는 factor 가 더 있을 때에는 ANCOVA 분석을 시행한다.

18.6.3 베이스라인 대비 6, 12주 시점에서 목표혈압에 도달한 비율

베이스라인 대비 6, 12주 시점에서 목표혈압에 도달한 비율에 대하여 기술통계량(빈도와 백분율)을 치료군별로 제시하고, 세 군간의 동질성에 대해 Pearson’s chi-square test 또는 Fisher’s exact test를 이용하여 분석한다.

18.6.4 2주 대비 12주째의 msSBP 및 msDBP 변화량

msSBP 및 msDBP에 대하여 2주, 12주 결과의 변화량에 대한 기술통계량(관측 대상 수, 평균, 표준편차, 중앙값, 최소값, 최대값)을 치료군별로 제시하고, 평균 변화량에 대한 세 군간의 비교는 ANOVA 또는 Kruskal-Wallis test를 이용하여 분석한다. 각 군내 변화는 Paired t-test 또는 Wilcoxon signed rank test를 통해 분석한다. 영향 주는 factor 가 더 있을 때에는 ANCOVA 분석을 시행한다.

# **19. 부작용을 포함한 안전성의 평가기준, 평가방법 및 보고방법**

이상 반응에 대한 평가는 3개월의 연구 기간 동안 각 방문 시마다 평가, 기록할 예정이며 환자와의 면담 및 직접적인 질문 등을 통해 시행한다. 안전성에 대한 평가는 이상 반응에 대한 자료 수집 (이상 반응 빈도, 심각도, 부작용의 종류, 심각한 이상 반응, 병용 약물, 신체 검사 결과, 활력 징후 측정, 심전도 측정 및 혈액 검사 등)을 통해 이루어지며, 환자가 호소하는 증상 및 징후 또한 연구 기간 동안 수집 및 기록될 것이다. 만약 임상시험 중 이상반응 발생 시, 연구자의 판단에 따라 시험의 중단 혹은 지속 유무를 결정할 것이며 이상반응으로 인한 결과가 해결되는 시점까지 추적 모니터링을 시행할 것이다.

19.1 이상반응의 정의

'이상반응 (Adverse Event, AE)'이란 임상시험용 의약품을 투여한 시험대상자에게 발생한 모든 유해하고 의도하지 않은 증후 (sign, 실험실 실험 결과의 이상 등을 포함한다), 증상 (symptom) 또는 질병을 말하며, 해당 임상시험용 의약품과 반드시 인과관계를 가져야 하는 것은 아니다.

'이상약물반응 (Adverse Drug Reaction, ADR)'이란 임상시험에 사용되는 의약품의 임의 용량에서 발생한 모든 유해하고 의도되지 않은 반응으로서 임상시험용 의약품과의 인과관계를 부정할 수 없는 경우를 말한다.

'중대한 이상반응 (Serious AE/SAE)'이란 임상시험에 사용되는 의약품의 임의의 용량에서 발생한 이상반응 중에서 다음 어느 하나에 해당하는 경우를 말한다.

- 사망하거나 생명에 대한 위험이 발생한 경우

- 입원할 필요가 있거나 입원 기간을 연장할 필요가 있는 경우

- 영구적이거나 중대한 장애 및 기능 저하를 가져온 경우

- 태아에게 기형 또는 이상이 발생한 경우

'예상하지 못한 이상약물반응 (Unexpected Adverse Drug Reaction)'이란 임상시험자 자료집 또는 의약품의 첨부 문서 등 이용 가능한 의약품 관련 정보에 비추어 이상약물반응의 양상이나 위해의 정도에서 차이가 나는 것을 말한다.

19.2 안전성 평가대상

임상시험용 의약품을 적어도 1회 이상 복용한 시험대상자에 한하여 실시한다.

19.3 평가기준 및 방법

시험대상자 개인별 이상반응 기록표에 기재된 이상반응 빈도, 정도 등과 실험실적 검사, 혈압 및 맥박의 활력징후결과에서의 이상소견을 고려하여 평가한다.

실험실적검사의 이상치, 활력징후 및 신체검사의 이상소견 중 임상적인 유의성이 있는 경우에는 증례기록서의 이상반응 기록표에 기재한다.

19.3.1 이상반응에 대한 평가

이상반응 발생여부, 증상, 발현일, 결과, 소실일, 중증도, 임상시험용 의약품과의 관련성, 관련된 조치, 치료 등 확인된 사항들을 증례기록서(case report form)에 기재한다.

19.3.1.1 임상증상 이상반응 정도에 대한 기준

① 경증(mild): 일상적인 활동을 제한하지 않는 정도로 시험대상자는 불쾌감을 느낌

② 중등증(moderate): 일상적인 활동을 제한하거나 영향을 미치는 정도로 시험대상자는 불쾌감을 느낌

③ 중증(severe): 일상적인 활동을 불가능하게 하는 정도의 이상반응

19.3.1.2 임상시험용 의약품과의 관련성

시험자는 발생한 이상반응과 임상시험용 의약품과의 관련성을 시험대상자의 과거력, 건강상태, 투약시간, 복용상태 등 여러 측면에서 검토하여 판단한다.

① 관련성이 명백함(certain)

- 이 약을 투여하였다는 증거가 있는 경우

- 이 약의 투여와 이상반응 발생의 시간적 순서가 타당한 경우

- 이상반응이 다른 어떤 이유보다 이 약 투여에 의해 가장 개연성 있게 설명되는 경우

- 투여 중단으로 이상반응이 사라지는 경우

- 재투여(rechallenge, 가능한 경우에만 실시) 결과가 양성인 경우

- 이상반응이 이 약 또는 동일 계열의 이 약에 대해 이미 알려져 있는 정보와 일관된 양상을 보이는 경우

② 관련성이 많음(probable/likely)

- 이 약을 투여하였다는 증거가 있는 경우

- 이 약의 투여와 이상반응 발생의 시간적 순서가 타당한 경우

- 이상반응이 다른 이유보다 이 약의 투여에 의해 더욱 개연성 있게 설명되는 경우

- 투여 중단으로 이상반응이 사라지는 경우

③ 관련성이 의심됨(possible)

- 이 약을 투여하였다는 증거가 있는 경우

- 이 약 투여와 이상반응 발생의 시간적 순서가 타당한 경우

- 이 약의 투여가 다른 가능성이 있는 원인들과 같은 수준으로 이상반응에 기인한다고 판단되는 경우

- 투여중단으로(실시된 경우) 이상반응이 사라지는 경우

④ 관련성이 적음(unlikely)

- 이 약을 투여하였다는 증거가 있는 경우

- 이상반응에 대해 보다 가능성이 있는 다른 원인이 있는 경우

- 투여 중단 결과(실시된 경우)가 음성이거나 모호한 경우

- 재투여(rechallenge, 가능한 경우에만 실시) 결과가 음성이거나 모호한 경우

⑤ 관련성이 없음(none)

- 시험대상자가 이 약을 투여 받지 않은 경우 또는,

- 약물투여와 이상 반응 발생간의 시간적 순서가 타당하지 않은 경우 또는,

- 이상반응에 대해 다른 명백한 원인이 있는 경우

⑥ 평가 불가능(unassessable)

- 이상 반응에 대한 일부의 정보는 있으나 이 약과 관련성에 대한 평가를 할 수 없는 경우

19.3.1.3 임상시험용 의약품에 관련된 조치

① 증량(dose increase)

② 용량변화 없음(dose not changed)

③ 감량(dose reduced)

④ 일시적 중단(dose interrupted)

⑤ 영구적 중단(drug withdrawn)

⑥ 해당사항 없음(not applicable)

⑦ 불명(unknown)

19.3.1.4 약물 치료

① 이상반응에 대하여 약물 치료를 함.

② 이상반응에 대하여 약물 치료를 시행하지 않음.

19.3.1.5 결과

① 회복(해결)됨(recovered/resolved)

② 회복(해결)중 임(recovering/resolving)

③ 회복(해결)되지 않음(not recovered/not resolved)

④ 회복(해결)되었으나 후유증이 남음(recovered/resolved with sequelae)

⑤ 사망(death)

⑥ 알 수 없음(lost to follow up/unknown)

19.4 이상반응의 보고 방법

임상시험 기간 중 발생한 '중대한 이상반응'과 관련한 각 담당자의 의무는 다음과 같다.

19.4.1 임상시험 책임자의 의무

임상시험책임자는 임상시험 중 모든 중대한 이상반응이 발생한 경우 즉시(시험자가 알게 된 날로부터 업무일 기준 1일) 임상시험의뢰자 및 지원기관에 보고하고, 추후에 상세한 내용이 포함된 추가보고를 하여야 한다. 단, 예상치 못한 중대한 이상약물반응의 경우 임상시험의뢰자 및 임상시험심사위원회에 신속 보고하여야 한다. 사망 예를 보고한 경우 시험책임자는 임상시험의뢰자와 심사위원회에게 부검보고서(부검을 실시한 경우에 한함)와 사망진단서 등의 추가적인 정보를 제공하여야 한다.

19.4.2 임상시험 담당자의 의무

임상시험 담당자는 임상시험 중 중대한 이상반응이 발생한 경우 즉시 임상시험 책임자에게 보고하고, 추후에 상세한 내용이 포함된 추가 보고를 하여야 한다. 단, 예상치 못한 중대한 이상약물반응의 경우 임상시험의뢰자 및 임상시험심사위원회에 신속 보고 하여야 한다.

19.4.3 임상시험심사위원회의 의무

임상시험심사위원회는 예상치 못한 중대한 이상약물반응이나 시험대상자의 안전성이나 임상시험의 실시에 부정적인 영향을 미칠 수 있는 새로운 정보에 관한 사항이 발생한 경우 임상시험 책임자에게 필요한 조치를 하도록 한다.

19.4.4 임상시험 의뢰자의 의무

(1) 의뢰자는 보고받은 모든 중대한 이상반응을 기타 관련된 시험자, 심사위원회(시험책임자가 심사위원회에 보고하지 않았거나 보고한 사항을 변경할 필요가 있는 경우만 해당) 및 식품의약품안전처에 보고 받거나 알게 된 날로부터 15일 이내에 보고하여야 한다. 단, 사망을 초래하거나 생명을 위협하는 경우에는 이 사실을 보고 받거나 알게 된 날로부터 7일 이내에 보고하고, 이 경우에 상세한 정보를 최초 보고일로부터 8일 이내에 추가로 보고하여야 한다. 의뢰자가 이상약물반응 보고서를 제출 시에는 임상시험 책임자 또는 담당자로부터 받은 내용을 제출한다.

(2) 의뢰자는 위의 보고와 관련하여 추가적인 안전성 정보를 주기적으로 해당 이상반응이 종결(해당 이상약물반응의 소실 또는 추적조사의 불가)될 때까지 보고하여야 한다. 이때 시험자는 보고에 대한 자료와 정보를 제공하는데 적극 협조하여야 한다.

(3)의뢰자는 지원기관인 한국메나리니㈜의 담당자에게도 보고하도록 한다.

임상시험의뢰자 연락처

고려대학교 구로병원 박창규

주소: 서울 구로구 구로동길 97, 고대구로병원 심혈관센터

전화 번호: 02)2626-3019

지원기관 연락처

한국메나리니㈜, PV 담당자 이연우

주소: 서울시 강남구 테헤란로 411 성담빌딩 12층

전화번호: 02)2037-7393

팩스: 02)2037-7373

이메일: yeonwoo.lee@menariniapac.com

19.5 이상반응 분석방법

치료군 별 약물 투여 후 발생한 이상반응, 이상약물반응(ADR), 중대한 이상반응(SAE)에 대하여 치료군별로 기술통계량(발현 대상자수, 발현율 및 발현 건수)과 발현율에 대한 95% 신뢰구간을 제시하고 치료군간 발현율 차이는 Pearson’s chi-square test 또는 Fisher’s exact test를 이용하여 비교 분석한다.

그외 실험실 검사치 및 활력징후 등에 대하여 결과치가 연속형 변수인 경우 약물 투여 전 대비 약물 투여 12주 후 변화량에 대해 치료군별로 기술통계량(관측 대상 수, 평균, 표준편차, 중앙값, 최소값, 최대값)을 제시하고 ANOVA 또는 Kruskal-Wallis test를 이용하여 분석한다. 각 군내 변화는 Paired t-test 또는 Wilcoxon signed rank test를 통해 분석한다. 결과가 범주형 변수인 경우, 약물 투여 전후 변화에 대한 분할표를 제시하고 군내 변화는 McNemar’s test를 이용하여 분석하고, 군간 차이는 Pearson’s chi-square test 또는 Fisher’s exact test를 이용하여 분석한다.

# **20. 임상시험 후 시험대상자의 진료 및 치료기준**

임상시험을 종료(조기종료 포함)한 시험대상자에 대한 시험 이후 진료 및 치료는 일상적인 진료 및 치료기준과 원칙에 따른다.

# **21. 시험대상자의 안전보호에 관한 대책**

본 연구는 임상시험심사위원회/기관생명윤리위원회에서 본 연구의 윤리적, 법적 요건을 충분히 검토하여 승인한 임상시험계획서에 의하여 시행되며, 또한 시험의 전 과정에 걸쳐 임상시험 관리기준 및 헬싱키 선언의 근본정신을 준수하게 될 것이다. 본 연구 도중에 시험대상자에 인권에 침해가 발생했을 경우 임상시험심사위원회/기관생명윤리위원회에 통보될 것이다.

시험대상자의 신원을 파악할 수 있는 정보는 연구자에 의해 비밀로 보장될 것이며, 이니셜과 코드화된 시험대상자식별정보로 연구 자료가 기록될 것이다. 또한 시험대상자 및 연구정보는 접근 제한된 컴퓨터에 저장될 것이며 시험대상자의 신상을 보호하기 위해 독립된 공간에서 연구에 대해 설명하고 동의서를 취득할 것이다. 임상시험의 결과가 출판될 경우에도 시험대상자 신상정보는 비밀상태로 유지될 것이다.

연구자는 본 임상시험에 참여한 연구대상자가 임상시험 참여 후 중대한 이상반응의 발생 등으로 응급실을 방문하거나 연구자의 판단에 따라 응급상황이라고 판단될 경우 즉시 임상시험을 중단하고 필요한 의학적 처리를 하여야 한다. 또한, 연구자의 판단에 따라 환자의 비정상 상태가 해소되거나 안정화되거나 더 이상 의학적인 처치가 필요하지 않을 때까지 추적 관찰해야 한다

# **22. 그 밖에 임상시험을 안전하게 과학적으로 실시하기 위하여 필요한 사항**

22.1 시험대상자 동의

시험자는 환자 및 그들의 보호자에게 본 임상시험에 관련된 모든 사항을 자세히 설명하고 모든 예측 가능한 결과에 대해 알 수 있는 충분한 기회를 주어야 한다. 환자가 동의한 내용은 반드시 문서로 기록되어야 한다.

시험자는 시험대상자 동의서에 서명하여 확인을 해야 한다. 시험자는 환자로부터 동의를 얻을 때까지는 임상시험만을 목적으로 한 특정 검사를 해서는 안 된다.

22.2 비밀보장

시험대상자의 이름은 모두 비밀로 유지하고 임상시험에서 부여한 번호에 의해 기록 및 평가 시 시험대상자를 확인한다. 시험대상자에게 모든 임상시험 자료가 엄격히 비밀사항으로 다루어진다는 것을 알려준다. 서명을 받은 동의서는 시험책임자가 보관한다. 본 계획서에 서명함으로써 시험책임자는 임상시험에 참가하는 시험대상자로부터 올바르게 동의서를 획득하기로 동의한 것이며 요청이 있는 경우 실사를 받는 데에도 동의한 것이다. 임상시험 책임자는 시험대상자번호 및 시험대상자명이 기록된 리스트를 갖추어 놓아 나중에 기록을 찾을 수 있도록 한다. 시험대상자 동의서와 대상자 리스트는 임상시험 완료일로부터 3년간 보관한다.

22.3 임상시험 계획의 숙지

시험책임자 및 담당자들은 임상시험계획을 정확히 분석 및 숙지하고, 임상시험을 실시한다.

22.4 임상시험계획서의 수정

임상시험 책임자의 동의 없이는 시험 도중 본 시험계획서의 내용을 변경 할 수 없다. 일단 시험이 시작된 후에는 예외적인 경우에만 수정을 해야 한다. 계획서 내용을 변경하는 경우에는 모든 관련 당사자들이 서명하여 서면으로 동의를 해야 한다. 수정된 내용은 임상시험심사위원회의 승인을 받아야 한다.

22.5 증례기록서(Case Report Form, CRF)

근거문서(source documents)라 함은 시험 기관에 보관되는 담당의사의 환자기록을 의미한다. 대부분의 근거문서는 병원이나 담당의사의 차트이며 환자의 증례기록서에 기록된 모든 정보는 해당 근거문서와 일치해야 한다.

자료의 입력 및 수정은 연구책임자로부터 권한을 위임받은 연구담당자가 하며, 자료의 최종 검토 및 서명은 연구책임자가 한다. 연구책임자는 서명을 통하여 증례기록서에 기록된 정보가 사실임을 보증하며 모든 경우에 있어서 증례기록서에 기록된 정보의 정확성 및 신뢰성에 대한 최종 책임을 갖는다.

증례기록서에 기입한 내용을 수정하는 경우에는 원 기록을 알아볼 수 있도록 해야 하고 수정한 임상시험담당자의 서명을 기재해야 한다.

22.6 모니터링 및 점검

임상시험이 KGCP에 따라 실시되고 시험자료가 국내외에서 등록 시 인정될 수 있도록 하기 위하여 연구책임자의 관리감독 하에 모니터링 및 점검을 실시할 수 있다.

본 임상시험의 자료 안전 점검자로서 연구책임자의 감독하에 증례기록이 완전하고 명확한지의 확인을 위하여 연구계획서에 따라 연구자료가 증례기록서에 수집되었는지 확인하고 기록원부와 대조 검토하여 자료의 완전성을 보증하고 대상자의 안전성 자료를 검토한다. 모니터링 시기는 적어도 최초 등록 시, 1년 마다 실시하도록 한다.

22.7 자료의 수집 및 관리

22.7.1 임상시험 결과의 기록

본 임상시험 중 수집된 모든 자료는 증례기록서에 기록하고 원본을 보관해야 한다.

연구자는 시험대상자의 정보 및 임상검사 결과 등 자료가 발생한 시점에 즉시 증례기록서에 기록한다.

자료가 누락된 경우에는 연구자가 타당한 설명을 붙여야 한다.

기록이 끝난 증례기록서는 연구책임자가 최종 서명한다.

22.7.2 임상시험 자료 관리

본 임상시험에서 사용하는 증례기록서의 개발, 유지 및 자료 관리는 의뢰자가 수행한다. 증례기록서에 기록된 임상자료의 입력 및 관리는 의뢰자가 지정한 별도의 담당자가 관리하며, 의뢰자는 데이터의 완전성, 정확성 및 일치성을 최종 관리해야 한다. 임상자료의 입력이 완료되면, 데이터 점검을 실시하여 데이터의 완결성, 정확성 및 신뢰성을 보장할 수 있도록 한다. 임상시험 데이터 관리를 위해 사용된 데이터베이스는 시스템 오류 또는 재난 등으로 인한 데이터 손실을 방지하기 위해 정기적인 백업을 실시하여 복구가 가능하도록 관리한다.

22.7.3 임상시험 결과의 이용

본 임상시험계획서에 서명함으로써 시험담당자는 본 시험의 결과를 등록, 발표 및 의약학 전문가들을 위한 정보제공 등의 목적으로 사용하는데 동의하는 것이다. 본 임상시험의 결과를 학술잡지 또는 학회지에 발표하기 전에 임상시험의뢰자는 발표내용을 검토할 권리가 있다.

22.7.4 자료의 보관

증례기록서의 원본 및 연구와 관련된 모든 자료 및 문서는 임상시험종료일로부터 3년간 보관해야 한다.

# **23. 참고문헌**

1. Schachinger V, Britten MB, Zeiher AM. Prognostic impact of coronary vasodilator dysfunction on adverse long-term outcome of coronary heart disease. Circulation. 2000;101:1899-1906

2. Lerman A, Zeiher AM. Endothelial function: Cardiac events. Circulation. 2005;111:363-368

3. Panza JA, Quyyumi AA, Brush JE, Jr., Epstein SE. Abnormal endothelium-dependent vascular relaxation in patients with essential hypertension. The New England journal of medicine. 1990;323:22-27

4. Taddei S, Virdis A, Mattei P, Salvetti A. Vasodilation to acetylcholine in primary and secondary forms of human hypertension. Hypertension. 1993;21:929-933

5. Rees DD, Palmer RM, Moncada S. Role of endothelium-derived nitric oxide in the regulation of blood pressure. Proceedings of the National Academy of Sciences of the United States of America. 1989;86:3375-3378

6. Suwaidi JA, Hamasaki S, Higano ST, Nishimura RA, Holmes DR, Jr., Lerman A. Long-term follow-up of patients with mild coronary artery disease and endothelial dysfunction. Circulation. 2000;101:948-954

7. Yasue H, Omote S, Takizawa A, Nagao M. Coronary arterial spasm in ischemic heart disease and its pathogenesis. A review. Circulation research. 1983;52:I147-152

8. Cockcroft JR, Chowienczyk PJ, Brett SE, Chen CP, Dupont AG, Van Nueten L, Wooding SJ, Ritter JM. Nebivolol vasodilates human forearm vasculature: Evidence for an l-arginine/no-dependent mechanism. The Journal of pharmacology and experimental therapeutics. 1995;274:1067-1071

9. Tzemos N, Lim PO, MacDonald TM. Nebivolol reverses endothelial dysfunction in essential hypertension: A randomized, double-blind, crossover study. Circulation. 2001;104:511-514

10. Dessy C, Saliez J, Ghisdal P, Daneau G, Lobysheva, II, Frerart F, Belge C, Jnaoui K, Noirhomme P, Feron O, Balligand JL. Endothelial beta3-adrenoreceptors mediate nitric oxide-dependent vasorelaxation of coronary microvessels in response to the third-generation beta-blocker nebivolol. Circulation. 2005;112:1198-1205

11. Maffei A, Lembo G. Nitric oxide mechanisms of Nebivolol
