# Supplementary material for: Comparison of nebivolol versus diltiazem in improving coronary artery spasm and quality of life in patients with hypertension and vasospastic angina: A prospective, randomized, double-blind pilot study
Source: PLoS One. 2020 Sep 11;15(9):e0239039. doi: 10.1371/journal.pone.0239039 (PMC7485806; doi:10.1371/journal.pone.0239039)
Supplement: S3 File — (DOCX) [file pone.0239039.s005.docx]

|  | **Clinical Trial Proposal** |
| --- | --- |

| **A pilot study for the Effect of Nebivolol on Coronary Arterial Spasm in Patients with Hypertension**  (investigator initiated clinical trial) |
| --- |

**Clinical Trial Protocol NO. NEB-VAS-01**

**Version 1.5**

| All information contained in this clinical trial protocol is provided to the principal investigator, clinical investigator, and clinical trial review committee. Written consent to participate in the trial is given to the person receiving the drug used in the clinical trial. It may not be disclosed to third parties without prior written consent, except in order to receive. |
| --- |

**[Overview of Clinical Trial Plan]**

| **Clinical Trial**  **Title** | A pilot study for the Effect of Nebivolol on Coronary Arterial Spasm in Patients with Hypertension |
| --- | --- |
| **clinical trials**  **Medicines** | Neviret tablets (Nebivolol hydrochloride)  Herben SR tablets (Diltiazem hydrochloride) |
| **Purpose** | Coronary spasm between nebivolol alone, diltiazem alone or low-dose nebivolol / diltiazem combination therapy in hypertensive patients with vasoconstrictive angina and check the effect on lowering blood pressure |
| **tests designed** | a prospective, randomized, double-blind trial, and the preliminary (Pilot) trial |
| **target disease** | a hypertension accompanied by vasospastic angina |
| **subjects** | 45 people (each group 15 people) |
| **selected / Exclusion**  **Criteria** | Selection Criteria  1) Diagnosing vasoconstrictive angina through coronary angiography and vasoconstriction testing among patients with hypertension (stage I-2: systolic blood pressure 140-179 mmHg and diastolic blood pressure 90-109 mmHg) between the ages of 20 and 80 Now the hospital treatment received patients as  possible.2) present decision to participate in the trial and voluntarily a party signing the  excluded based on  agreement:1) history showed hypersensitivity to beta-blockers or calcium antagonists  or the2) dementia or associated psychiatric disorders History of abuse of drugsThose  3)who participated in other clinical trials within one month prior to screening  4) Those who judged the investigator to be in a medical condition incapable of implementing the procedures and procedures of the protocol or inadequate for participation  5) Influencing the efficacy evaluation of the study drug Subjects who are taking drugs that can give drugs (ACE inhibitors, angiotensin blockers, beta blockers other than clinical trial drugs, calcium antagonists other than clinical trial drugs, diuretics other than indapamide) (these subjects are washed for at least 2 weeks) out period after possible participation)  6) malignant hypertension (retinal bleeding, or if there is papilledema) or known malicious or moderate retinopathy (retinal bleeding within the last 6 months, visual acuity Disorders, retinal microaneurysm)  7) History of secondary hypertension and all suspected secondary hypertension: aortic stenosis, hyperaldosteronemia, renal artery stenosis, Cushing's disease, chromocytoma, polycystic kidney disease, etc.  8) orthostatic with symptoms Patients with hypotension  9) People with severe heart disease (heart failure (NYHA class 3 and 4), ischemic heart disease (angina pectoris, myocardial infarction), percutaneous coronary dilatation, or coronary artery bypass surgery in the last 6 months)  10) Severe Patients with cerebrovascular disorders (stroke, cerebral infarction, cerebral hemorrhage, etc. within the last 6 months)  11) Urinary or severe renal failure (creatinine clearance <30mL / min)  12) Severe liver failure or AST or ALT> 3 times normal upper limit, biliary atresia Patients with biliary cirrhosis, cholestasisPatients  13)with gastrointestinal disease and surgery that may affect the absorption, distribution, metabolism, and excretion of the drug, current active gastritis and gastrointestinal / rectal bleeding, which the investigator deems clinically significant, recent Active inflammatory bowel syndrome within 12 months, etc.  14) Pregnant and lactating women, women of childbearing potential who have a pregnancy plan or do not agree with an appropriate method of contraception during the clinical trial  * Suitable methods of contraception are as follows.  (1) Progestin monotherapy hormone therapy (oral, transplanted tablets)  (2) Intrauterine device  (3) Blocking method: spermicide and condom / spermicide and closed cap (contraceptive diaphragm or cervical / palatal cap)  (4)theof male vasectomy |
| **methodapply** | as randomly assigned such that a ratio of three groups:this study vascular contractility angina is a group as a preliminary (pilot) test to evaluate the efficacy and safety of the navigation borol in hypertensive patients 1 with 1 We want to compare the progress and results by dividing.  ◇tablet) 5mg / day (2 weeks) → 10mg / day (10 weeks)  Nebiborol group (Group 1): Oral nebiborol (Naviret◇ Diltiazem group (Group 2): Oral diltiazem (Herben) Sustained release) 90mg / day (2 weeks) → 180mg / day (10 weeks)  ◇ Nebivolol + Diltiazem group (Group 3): Oral Nebivolol (Naviret tablet) 2.5mg / day + Oral Diltiazem (Her Ben sustained-release tablet) 45mg / day (two weeks) → oral Guinea borol (Guinea inlet tablets)5mg / day + oral diltiazem (Herr Ben sustained-release tablet) 90mg / day (10 weeks)forfor  toclinical trial drug in each perioda mealthe Regardless, it is taken orally once a day in the morning. |
| **Evaluation periodtest period** | for 2 years from the IRB approval date of the |
| **and**  **evaluation method** | 1. Primary efficacy endpoint  (1) Change in msSBP and msDBP atChange  week 12 compared to baseline (2)in quality of angina at week 12 compared to baseline  2. Secondary efficacy endpoint  ( 1)coronary spasm at week 12 compared to baseline  Changes in(2) Changes in msSBP and msDBP at week 6 compared to baseline  (3) Ratios of reaching target blood pressure at weeks 6 and 12 at baseline  (4) Week 12 compared to week 2 msSBP and msDBP changes  (target blood pressure *: msSBP / DBP <140 / 90mmHg)  3. Safety evaluation variables  adverse reactions, laboratory tests, physical examination, vital signs (pulse), ECG |
| **statistical analysis** | 1. Primary evaluation variables  1) Baseline The amount of change inweek 12 compared to that of  msSBP and msDBP atmsSBP and msDBP, descriptive statistics (number of observations, average, standard deviation, median, minimum, maximum) for baseline and 12-week results are presented for each treatment group and Comparisons between the three groups were analyzed using ANOVA or Kruskal-Wallis test. Changes within each group are analyzed by paired t-test or Wilcoxon signed rank test. When there are more factors influencing, ANCOVA analysis is performed.  2)quality of change in the  Compare the average of the total scores that were added through the baselineangina at week 12 and the Seattle angina questionnaire at week 12 compared to the baseline. The comparison between the three groups for the mean change was analyzed using ANOVA or Kruskal-Wallis test. Changes within each group are analyzed by paired t-test or Wilcoxon signed rank test. When there are more factors influencing, ANCOVA analysis is performed.  2. Secondary evaluation variables  1)week 12 compared12 weeks for coronary spasm  Change in coronary spasm atto baseline Treatment group treated with descriptive statistics (number of observations, mean, standard deviation, median, minimum, maximum) for changes in baseline andThe results are presented separately, and comparisons between the three groups for the average change are analyzed using ANOVA or Kruskal-Wallis test. Changes within each group are analyzed by paired t-test or Wilcoxon signed rank test. When there are more factors influencing, ANCOVA analysis is performed.  2)Week 6 compared  Changes in msSBP and msDBP atto Baseline Descriptive statistics (number of observations, average, standard deviation, median, minimum, maximum) for the change in baseline and 6 weeks results for each treatment group , Comparison between the three groups for the average change was analyzed using ANOVA or Kruskal-Wallis test. Changes within each group are analyzed by paired t-test or Wilcoxon signed rank test. When there are more factors influencing, ANCOVA analysis is performed.  3) Proportion of reaching target blood pressure at  6 and 12 weeks compared to baseline The descriptive statistics (frequency and percentage) are presented for each treatment group for the ratio of reaching target blood pressure at 6 and 12 weeks compared to baseline, and between the three groups Homogeneity is analyzed using Pearson's chi-square test or Fisher's exact test.  4)week 12 compared to week  Changes in msSBP and msDBP at2 Describe the statistical statistics (number of observations, average, standard deviation, median, minimum, and maximum) for each treatment group for msSBP and msDBP. Then, the comparison between the three groups for the average change was analyzed using ANOVA or Kruskal-Wallis test. Changes within each group are analyzed by paired t-test or Wilcoxon signed rank test. When there are more factors influencing, ANCOVA analysis is performed.  3. Safety evaluation variables  95% confidence in descriptive statistics (number of subjects, expression rate and number of expressions) and expression rate for each treatment group for adverse reactions, adverse drug reactions (ADRs), and severe adverse events (SAEs) after drug administration by treatment group The intervals are presented and the differences in expression rates between treatment groups are compared and analyzed using Pearson's chi-square test or Fisher's exact test.  If the results of other laboratory tests and vital signs are continuous variables, descriptive statistics (number of observations, average, standard deviation, median, minimum, maximum) for each treatment group for the amount of change 12 weeks after drug administration compared to before drug administration And analyzed using ANOVA or Kruskal-Wallis test. Changes within each group are analyzed by paired t-test or Wilcoxon signed rank test. When the results are categorical variables, a division table for changes before and after drug administration is presented, and intragroup changes are analyzed using McNemar's test, and differences between groups are analyzed using Pearson's chi-square test or Fisher's exact test. |

**[Clinical trial schedule]**

| Schedule | Visit 1 | Visit 2^a^ | Visit 3 | Visit 4 | Visit 5 |
| --- | --- | --- | --- | --- | --- |
| Week (Day) | Week -4 ~ | Baseline  (Day 0) | Week 2  (Day 14)+5 | Week 6  (Day 42)+5 | Week 12  ( Day 84)+14 |
| Acquisition of consent | ● |  |  |  |  |
| Confirmation of selection / exclusion criteria | ● |  |  |  |  |
| Demographic survey | ● |  |  |  |  |
| Medical history survey | ● |  |  |  |  |
| Weight^b^, blood pressure and heart rate measurement | ● | ● | ● | ● | ● |
| Pregnancy test | ●^c^ |  |  |  |  |
| randomization |  | ● |  |  |  |
| Clinical trial drug prescription |  | ● | ● |  |  |
| Coronary angiography And vasoconstriction test | ●^d^ |  |  |  | ● |
| Seattle angina questionnaire survey |  | ● |  |  | ● |
| Prior^e^/ concomitant medication check | ● | ● | ● | ● | ● |
| Adverse reaction check |  | ● | ● | ● | ● |
| Return drug return and compliance assessment |  |  | ● |  | ● |

a. Screening and baseline visits and procedures can be performed on the same day if screening / exclusion criteria can be evaluated during screening.

b. Body weights are only examined at screening and visit 5.

c. Women of childbearing potential with a positive pregnancy test cannot participate in clinical trials.

d. It is necessary for daily medical examination before participation in the study, and is recruited for patients who have undergone the examination. Test values ​​within 4 weeks prior to screening are available.

e. The preceding drug should be investigated only at screening.

**Contents**

[**1. Names and stage of clinical trials,** 8](#_1fob9te)

[**clinical trials Organization2.and address,** 8](#_3znysh7)

[**3. Namejob title and responsibilities of a clinical trial, researchers, staff, co-workers** 8](#_2et92p0)

[**4. clinically apparent and commission Addresstest** 8](#_tyjcwt)

[**5. Clinical trial research, Clinical trial yogurt product support institution name and address** 8](#_3dy6vkm)

[**6. Purpose and background of the** 8](#_1t3h5sf)

[**clinical trial7. Code name of clinical trial drug (or generic name of the main ingredient), drug substance and quantity, formulation, etc. (if applicable)** 10](#_4d34og8)

[**8. Drugs for clinical trials** 10](#_2s8eyo1)

[**9. Diseases to be tested** 11](#_17dp8vu)

[**10. Selection criteria and exclusion criteria for** 11](#_3rdcrjn)

[**subjects11. Number of target subjects and their basis** 12](#_26in1rg)

[**12. Duration of** 12](#_lnxbz9)

[**clinical trials13. Methods of clinical trials** 12](#_35nkun2)

[**14 Observation items, clinical test items, and observation test methods** 13](#_1ksv4uv)

[**15. Predictive adverse reactions and precautions for use** 16](#_44sinio)

[**16. Plan violation, suspension and dropout criteria** 24](#_2jxsxqh)

[**17. Evaluation variables** 24](#_z337ya)

[**18. Evaluation criteria, evaluation methods and interpretation methods (statistics) Analytical methods)** 24](#_3j2qqm3)

[**19. Evaluation criteria, evaluation methods and reporting methods for safety including side effects** 26](#_1y810tw)

[**20. Treatment and treatment standards for subjects after clinical trials** 30](#_4i7ojhp)

[**21. Measures for safety protection of subjects** 30](#_2xcytpi)

[**22. Other clinical trials Necessary to safely and scientifically conduct the** 31](#_1ci93xb)

[**23. References** 32](#_3whwml4)

testSubject Description and Consent: Appendix 1

Conventions for Compensation for Victims: Appendix 2

Participants in Clinical Trials: Appendix 3

# **1. Names and Stages of Clinical Trials**

A pilot study for the Effect of Nebivolol on Coronary Arterial Spasm in Patients with Hypertension

# **2. Clinical trial institution name and address**

1) Korea University Guro Hospital Cardiovascular Center, 97 Guro-dong-gil, Guro-gu, Seoul

2) Circulatory Internal Medicine, Korea University Anam Hospital, 73 Inchon-ro, Seongbuk-gu, Seoul

3) Shinchon Severance Hospital, Yonsei Medical Center, Seoul Seo 50-1, Yonsei-ro

# **,3. Name and title of the principal investigator, researcher, and co-researcher**

3.1 Head of clinical trials

| guKorea University Guro Hospital | Chang-Gyu Park,/ |
| --- | --- |
| Anam Hospital, Korea University | Daemun-Senior Researcher / Professor, Department of Circulatory Medicine Hong Soon-Joon,/ Professor, Department of Circulatory Medicine |
| Severance Hospital, Yonsei Medical Center | Joong-sun Kim / Senior Researcher / Professor of Cardiology Department |

3.2 Clinical Trial Manager, Management Pharmacist, Coordinator

Attached 3. Refer to Clinical Trial Participant

# **4. Name and Address of Clinical Trial**

Park, Chang-Gyu Park, Professor, Korea University Guro Hospital Cardiovascular Center (Professor of Circulatory Internal Medicine), Seoul, Korea 97, Guro-dong-gil, Guro-gu

# **5. Institutional ResearchInstitutional Drug Supporting Organizations**

Funds andMenarini Korea, 411, Teheran-ro, Gangnam-gu, Seoul, 12th floor, Seongdam Building

# **6. Purpose and Background of the Clinical Trial**

endothelial dysfunction; The correlation between nitric oxide function impaired in vascular endothelial cells and the risk of coronary artery disease is well known through previous studies.^1,2 The^ deterioration of nitrogen monoxide (NO) functioning on the vascular lining is mainly explained by synthetic degradation, loss due to oxidative stress, and reduced sensitivity to vasodilation.^3,4 In^ particular, hypertensive patients have been known to develop vascular endometrial dysfunction through animal experiments and several clinical studies, mainly due to an increase in biomechanical friction in blood vessels and a decrease in the bioavailability of nitrogen monoxide, which in the end This leads to mismatch of nitric oxide production in the vascular lining and changes in normal vasodilation capacity.^5^ Recently, there have been reports that early diagnosis and treatment for endometrial dysfunction can improve endometrial dysfunction and prevent the progression of coronary artery disease.^6^ However, it is a reality that the drugs to be selected in patients with vasoconstrictive angina with impaired endometrial function are very limited. Until recently, beta-blockers have been reported to inhibit vasodilator action on adrenaline stimulation, making them a relatively contraindicated agent in patients with vasoconstrictive angina. In one study, propranolol not only inhibited vasoconstrictive angina, but rather exacerbated it. There was a report.^7^ However, recently the third generation beta blocker developed (eg Kaveh carvedilol (carvedilol), Guinea borol (nebivolol)) itttareumyeonseo are looking for the vasodilating action of which the role of beta-blockers in vascular contractility angina is revisited, in particular, , Nebivolol (selective, long-acting beta-blocker) is known to block β-1 adrenergic receptors and act on vasodilation by stimulating β-3 adrenergic receptors. It is also known to have an effect-inducing effect. (Figure)^8-11^ Therefore, this clinical trial piloted assuming that nebivolol inhibits vasoconstriction in hypertensive patients and will be effective in patients with vasoconstrictive angina. I want to proceed with the test.
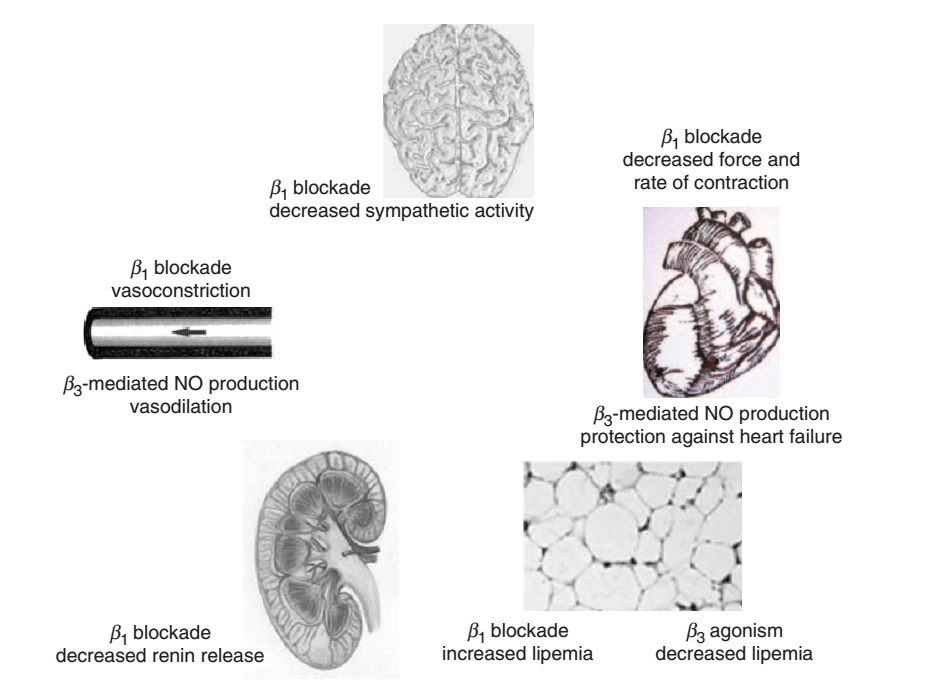


Drawing. Mechanism and effect of Nebivolol on major organs.^11^ Nebivolol selectively inhibits β-1 adrenergic receptors and suppresses vasoconstriction. In particular, it increases with the effect of stimulating β-3 adrenergic receptors in the heart.

# **7. Code name of clinical trial drug (or generic name of main ingredient), drug substance and its quantity, formulation, etc. (if applicable)**

1) Nebiret tablets 5mg

(1) Product name: Nebiret tablets (Neviborol hydrochloride)

(2 ) Formulation and Properties: White round tablet

(3) Content: Nebirolol hydrochloride 5.45mg (Neviborol 5mg)

(4) Storage method: Airtight container, room temperature (1-30 ℃) storage

2) Herbenserine 90mg

(1) Product Name: Herbenserine (Diltiazem Hydrochloride)

(2) Formulation and Properties: White to milky white sustained release

(3) Content: Diltiazem Hydrochloride 90mg

(4) Storage: Airtight container, room temperature storage (1-30 ℃ )

# **8. Drugs for clinical trials**

8.1 Labels for drugs for clinical trialslabel

1) "Clinical trial"

2) Code name of product or generic name of main ingredient

3) Manufacturing number and use (validity) due date or retest date

4) Storage method

5) The name and address of the person who has been approved for the clinical trial plan.

6) The indication that "cannot be used for purposes other than a clinical trial"

8.2 Issuance, storage, management, and recording of the

trial drug The investigator receives, transports and status of the drug used in the trial. And record the receipt, delivery and return of the medication used in the clinical trial.

1) Dispensingissuance

of medicines used forclinical trials should be done by a prescription signed by the investigator or the investigator participating in this clinical trial.

2) Storage, management, and records

Clinical trial drugs should be received and secured and handled and stored by a designated person at each clinical trial institution and stored in a safe place accessible only by the designated person. After receipt, the drug should be stored according to the instructions on the label for the investigational drug.

A pharmacist who manages clinical trials should record and manage the subject's assignment number, prescription date, and amount of dispensation on the ledger of medicines, identify the status of use, and keep a list of receipts, grants, and returns of medicines used in clinical trials. .

The investigator should not use the investigational drug and related products for purposes other than those specified in the clinical trial protocol.

3) Return of unused drugsreturn

Clinical trial management pharmacistsunused drugs to support organizations only for clinical trial drugs provided by the support institution. When a clinical trial institution discards or loses a clinical trial drug, the manager records such matters in a clinical trial drug management table. At the end of the clinical trial, all unused clinical trial drugs, including those used for clinical trials and return drugs, that have not been issued to the subject, must be returned to the supporting institution. When shipping medicines used in clinical trials, a shipping record confirming the medicines returned should be attached.

# **9.disease**

Patients with hypertension with vasoconstrictive angina

# **10. Selection criteria and exclusion criteria for test subjects**

10.1 Selection criteria

1) Hypertension between 20 and 80 years old (stage I-2: systolic blood pressure 140-179 mmHg and diastolic blood pressure) 90-109mmHg) Patients who have been diagnosed with vasoconstrictive angina through coronary angiography and vasoconstriction tests. Those who can go to hospital treatmentThose who

2)decide to participate in this clinical trial and voluntarily sign consent form

10.2 Exclusion Criteria

1) History of hypersensitivity to beta-blockers or calcium-blockersHistory of

2)dementia or accompanying psychiatric disorders or drug abuse

3) Participants in other clinical trials within 1 month prior to screening

4) Follow the protocol's compliance and procedures to implement or test self-determined as unsuitable medical conditions in the participant

5) study drug could affect the efficacy assessment about (ACE inhibitors, angiotensin blockers, investigational drug other beta-blockers, investigational drug other calcium antagonists, which of Indah Subjects who are taking diuretics (except for phamide) (these subjects can participate after at least 2 weeks of wash-out period)

6) Malignant hypertension (with retinal bleeding or papillary edema) or known moderate or malignant retinopathy (Retinal hemorrhage, visual impairment, retinal microaneurysm within the last 6 months)

7) History of secondary hypertension and all suspected secondary hypertension: aortic stenosis, hyperaldosteronemia, renal artery stenosis, Cushing's disease, chromocytoma, polycystic kidney disease Etc.

8) Patients with orthostatic hypotension with symptoms

9) People with severe heart disease (heart failure (NYHA class 3 and 4), ischemic heart disease (angina pectoris, myocardial infarction) within the last 6 months, percutaneous coronary artery dilatation, or coronary artery bypass surgery) party received such treatment),

10), cerebrovascular disorder patients with severe (last 6 months within a stroke, cerebral infarction, cerebral hemorrhage, etc.)

11) munyo or severe renal failure (creatinine clearance <30mL / min)

severe hepatic or AST or ALT> 3 times normal upper limit, biliary atresia, biliary cirrhosis, and cholestasis patientsPatients

13)with gastrointestinal diseases and surgery that may affect the absorption, distribution, metabolism, and excretion of drugs. Active gastritis and gastrointestinal / rectal bleeding, active inflammatory bowel syndrome within the last 12 months, etc.

14) Pregnant and lactating women, women of childbearing potential who have a pregnancy plan or do not agree with an appropriate method of contraception during the clinical trial

* Suitable methods of contraception are as follows.

(1) Progestin monotherapy hormone therapy (oral, transplanted tablets)

(2) Intrauterine device

(3) Blocking method: spermicide and condom / spermicide and closed cap (contraceptive diaphragm or cervical / palatal cap)

(4) Male Vascular Resection

# **11. The number of target subjects and their basis**

This study is a pilot study because there are no prior studies related to the study, and the patient group is divided into 3 groups. Will recruit.

# **12. Duration of clinical trialthe date of**

2 years fromIRB approval

# **13. Method of clinical trial**

13.1 Study design and method

This study is a pilot study to evaluate the efficacy and safety of nebivolol in hypertensive patients with vasoconstrictive angina. The ratio is randomly assigned to a 1: 1: 1 ratio and divided into 3 groups to compare progress and results.

◇tablet) 5mg / day (2 weeks) → 10mg / day (10 weeks)

Nebiborol group (Group 1): Oral nebiborol (Naviret◇ Diltiazem group (Group 2): Oral diltiazem (Herben) Sustained release) 90mg / day (2 weeks) → 180mg / day (10 weeks)

◇ Nebivolol + Diltiazem group (Group 3): Oral Nebivolol (Naviret tablet) 2.5mg / day + Oral Diltiazem (Her Ben sustained-release tablet) 45mg / day (two weeks) → oral Guinea borol (Guinea inlet tablets)5mg / day + oral diltiazem (Herr Ben sustained-release tablet) 90mg / day (10 weeks)forfor

toclinical trial drug in each perioda mealthe Regardless, it is taken orally once a day in the morning.

13.2 Method of randomization If

this clinical trial satisfies the selection criteria and the suitability of the subjects who agree to participate in the study is finally confirmed, the subjects are sequentially assigned random numbers according to the randomization table. The randomization table allows the ratio of each group to be assigned 1: 1: 1 by the block randomization method, and the randomization table is programmed using the random allocation software program, and takes into account the predetermined block size. Assign a random number of sizes.

13.3 Criteria for positive judgment of vasoconstrictive angina The

changes in coronary artery according to drug administration were analyzed with a computerized quantitative analyzer (QCA) by measuring the inside diameter of the artery with a caliper, and the value measured with a caliper was used as a reference value for the outer diameter of a catheter filled with contrast medium. As a result, the epicardial coronary artery is defined as having a local or overall significant change of 70% or more and chest pain, or an ischemic change on the electrocardiogram (ST segment rise or fall of 1 mm or more, T wave inversion). In addition, the vasoconstriction rate was defined as [(inner diameter after administration of nitroglycerin-inner diameter after administration of acetylcholine) / inner diameter X100 after administration of nitroglycerin].

13.4 Combination therapy and contraindications

Subjects may not be administered any other medications that may affect the outcome of the study, except for investigational medications during the trial period. However, in the case of SBP 140, DBP 90 or higher after 6 weeks of administration, indapamide (1.25 mg / day po) may be allowed, and if necessary, it may be allowed if it is determined to be a drug that does not affect the test results according to the investigator's judgment. Can be. The drug name, daily dose, purpose of administration, and duration of use of the combined drug must be recorded in the case record, regardless of whether or not allowed during the clinical trial period.

Contraindicated drugs:

- ACE inhibitors,
- angiotensin
- clinical trial drugs other beta blockers
- investigational drug other calcium antagonists
- blockers,exceptindazol Pharma Id diuretic

# **14. observation items, check items and the clinical observation test method**

14.1 clinical trials progress schedule

| calendar | 1Visit | Visit 2^a^ | 3 Visit | Visit 4 | Visit 5 |
| --- | --- | --- | --- | --- | --- |
| Week (Day) | Week -4 ~ | Baseline  (Day 0) | Week 2  (Day 14)+5 | Week 6  (Day 42)+5 | Week 12  (Day 84)+14 |
| Obtain consent form | ● |  |  |  |  |
| Confirm selection / exclusion criteria | ● |  |  |  |  |
| Demographics Survey | ● |  |  |  |  |
| Medical history survey | ● |  |  |  |  |
| Weight^b^, blood pressure and heart rate measurement | ● | ● | ● | ● | ● |
| Pregnancy test | ●^c^ |  |  |  |  |
| randomization |  | ● |  |  |  |
| Prescription for clinical trials |  | ● | ● |  |  |
| Coronary angiography and vasoconstriction induction | ●^d^ |  |  |  | ● |
| Seattle angina questionnaire survey |  | ● |  |  | ● |
| Prior^e^/ Confirmation of concomitant medication | ● | ● | ● | ● | ● |
| Adverse reaction confirmation |  | ● | ● | ● | ● |
| Returned drug recovery and compliance evaluation |  |  | ● |  | ● |

a. Screening and baseline visits and procedures can be conducted on the same day if screening / exclusion criteria can be evaluated during screening.

b. Body weights are only examined at screening and visit 5.

c. Women of childbearing potential with a positive pregnancy test cannot participate in clinical trials.

d. It is necessary for daily medical examination before participation in the study, and is recruited for patients who have undergone the examination. Test values ​​within 4 weeks prior to screening are available.

e. The preceding drug should be investigated only at screening.

14.2 Observation Items

14.2.1 Written consent and screening number are given

to patients who have undergone coronary angiography and vasoconstriction tests, who are deemed appropriate to participate in the study. Provide all information and obtain the subject consent form including the subject's name, signature, and date. Screening numbers are given in the order of obtaining the consent of the subjects. Screening numbers are given as follows.

- SXXZZZ (S: initial letter of screening, XX: institution number, ZZZ: serial number)

Subject consent must be reached prior to any clinical trial. The consent form must be recorded in handwritten name, signature, and date by the subject or legal representative, which means the subject's voluntary consent. In addition, the investigator of this clinical trial should record the name, signature, and date confirming the consent of the test subject together in the written consent of the test subject.

14.2.2 Assignment of registration number If the

test subject satisfies all the selection criteria in this study protocol and does not meet the exclusion criteria, the investigator assigns the registration number in the order of prescribing the investigational drug. At this time, the registration number is consistent with the order of treatment group assignment.

14.2.3 Demographic Investigation
The date and date of birth, gender, height, weight, waist circumference, hip circumference, and neck circumference of the subject who agreed in writing are investigated.

14.2.4 Medical history investigation
The medical history of the subjects who have been given a screening number is investigated. Investigate the existence of smoking, drinking history, family history of chronic diseases, past and present medical history.

14.2.5 Clinic BP and Pulse Measurement
Blood pressure and pulse are measured prior to other tests at the time of visit, and left blood pressure and pulse are measured when stable.

Blood pressure is measured for systolic and diastolic blood pressure twice every two minutes with both arms in a sitting position after resting for at least 5 minutes or more. If the diastolic blood pressure difference measured twice is more than 10 mmHg, measure it once more and record the average value of the 2nd and 3rd blood pressure. At the baseline visit, blood pressure is measured at the upper arm of both arms, and only the blood pressure of the arm with high blood pressure is measured thereafter.

14.2.6 Pregnancy test For

women of childbearing age, except for subjects with menopause or those undergoing fertility surgery, urine tests (urine-HCG) confirm pregnancy.

14.2.7 Coronary angiography and vasoconstriction prophylaxis

This study was conducted for patients diagnosed with vasoconstrictive angina through coronary angiography and vasoconstriction tests under routine treatment prior to participation in the study.

General coronary angiography and vasoconstriction-induced tests are performed as follows . :

The target patient stops medications that may affect the vasoconstriction-induced test ( eg nitrate, calcium channel blockers, beta blockers, etc.) 48 hours before the test. Coronary angiography is performed by puncturing the radial or femoral artery and inserting a Judkins catheter . An electrocardiogram is attached to record changes in the electrocardiogram during the triggering test before coronary angiography . Contrast agent is a non-ionized product, ultravist , and in order to induce vasoconstriction , dilute acetylcholine , a product manufactured by Tokyo's first pharmaceutical company , in physiological saline. The dose of acetylcholine is sequentially increased to the left coronary artery in the order of 10 ug (A1), 25 ug (A2), and 50 ug (A3), and then slowly injected over about 60 seconds, and each acetylcholine administration proceeds at 2 minute intervals. . When the induced test is over or significant coronary artery contraction, chest pain , and electrocardiographic changes occur after administration of acetylcholine drug , 200 ug of nitroglycerin is injected into the coronary artery .

14.2. 8 Seattle Angina Questionnaire This questionnaire was developed to be used as a means of determining the treatment effect of patients with ischemic heart disease and has the advantage of being able to specifically evaluate the quality of life associated with angina. The questionnaire is composed of 19 items in 5 categories, and it is a method of calculating and adding scores from 0 to 100 (best) for each category. In this study, a survey will be conducted before the start of drug treatment and at the end of treatment, 3 months.

14.2. 9 Preceding / concomitant drug identification

Prior drugs are investigated for drugs administered within 4 weeks prior to screening . For concomitant medications, concomitant medications administered since the previous visit should be investigated .

14.2. 10 adverse reaction evaluation

Evaluate and record the adverse reactions that occurred in the subjects after administration of the study drug.

When laboratory tests, electrocardiograms, and echocardiography tests are performed under the routine treatment of subjects outside the course of this study , the researchers refer to the test results and clinically significant results at the time of completion of administration compared to before the study drug administration Changes can be judged and evaluated as adverse reactions .

**15. Predictive adverse reactions and precautions for use**

All clinical trial drugs used in this clinical trial are commercially approved drugs, and the precautions for each use are as follows.

15.1 Precautions for using Naviret tablet

1. Do not administer to the next patient.

1) People with hypersensitivity to this drug

2) Patients with a history of bronchial spasm or bronchial asthma

3) patients with metabolic acidosis

4) Bradycardia (<60 bpm), 2 ~ 3 degree AV block patients

5) Patients with decompensated heart failure who require acute heart failure, cardiogenic shock or inotropic treatment (intravenous injection)

6) Uncontrolled deep transition

7) Patients with hypotension ( systolic blood pressure <90mmHg)

8) Patients with severe peripheral circulation disorder

9) Patients with dysfunction syndrome including oriental blockade

10) Patients with untreated chromium-friendly cell tumor

11) Patients with liver failure or impaired liver function

12) Pregnant or lactating women

13) Patients with chronic renal failure with severe renal failure (serum creatinine ≥2.5 mg / dL)

14) The drug because it contains lactose, galactose intolerance ((galactose intolerance), Lapp lactose intolerance (Lapp lactase deficiency), or glucose-patients with a genetic problems such as galactose malabsorption (glucose-galactose malabsorption) for Do not administer.

2. The next patient should be administered carefully.

1) Patients with chronic impaired lung disorder

2) Untreated congestive heart failure (CHF) patients

3) patients with ischemic heart failure

4) Patients with peripheral circulatory disorders ( Raino syndrome , intermittent claudication)

5) Patient with atrioventricular block of 1 degree

6) Prinzmetal angina pectoris due to non-resistance α-receptor-mediated coronary vasoconstriction

7) Thyroid Hyperthyroidism patients ( tachycardia symptoms to conceal can.)

8) Patients with a history of psoriasis

9) Diabetic patients (does not affect blood sugar levels, but can mask certain symptoms of hypoglycemia ( tachycardia , palpitations).)

10) Elderly people

3. Adverse reactions

Adverse reactions were divided into hypertension and chronic heart failure.

<Hypertension>

1) Hypersensitivity: A rash may appear.

2) Eyes: Sometimes visual impairment may occur.

3) Circulatory system: Sometimes bradycardia , AV blockage, AV conduction delay, hypotension, heart failure, and intermittent claudication (increase).

4) Mind nervous system: Frequent headaches, dizziness, sensory abnormalities, sometimes depression, nightmares, and very rarely fainting.

5) Digestive system: Diarrhea, constipation, nausea, indigestion, bloating, and vomiting may occur frequently.

6) Respiratory system: Frequent dyspnea, and sometimes bronchospasm.

7) Skin and subcutaneous tissue: Sometimes itching, erythematous rash, and very rarely exacerbation of psoriasis.

8) Other: Frequent edema, fatigue, and erectile dysfunction.

9) Immune system : Infrequent vascular edema and hypersensitivity may occur.

10) The following adverse reactions have been reported in other beta-blockers.

   ① Mental nervous system: hallucinations, psychosis, mixed mood, blue extremities

   ② Eyes: dry eye, practolol type eye mucosa skin toxicity

   ③ Others: Raynoid phenomenon

<Chronic heart failure>

1) Very often bradycardia , dizziness, frequent exacerbation of heart failure, orthostatic hypotension , drug intolerance , first-degree atrioventricular block , and edema of the lower extremities may occur.

< Results of domestic post-marketing investigation>

As a result of use survey conducted on 757 people for 4 years in Korea, the incidence of adverse events was 25.6% (194/757, 346 cases) regardless of causality .

-The incidence of adverse drug reactions that cannot be excluded from causality was 15.2 % (115/757 patients, 166 cases). Reported adverse drug reactions were dizziness in 2.8% (21 patients, 21 cases), headaches in 1.2% (9 patients, 9 cases), constipation, and edema 0.8% (6 patients, 6 cases), nausea, and heartburn. , Diarrhea, insomnia, palpitations 0.7% (5, 5 cases), dyspepsia, abdominal pain, bradycardia 0.5% (4, 4 cases), vomiting, urination , blood sugar rise 0.4% (3 persons) , 3 cases), neck / shoulder pain 0.3% (2 people, 3 cases), sensory abnormalities, gastritis, chest pain, fatigue, asthenia, lack of effect, weight loss, difficulty breathing, cough, cold, dry cough, anorexia , Anxiety, back pain , increase in blood creatinine , ringing 0.3% (2 people, 2 cases), dizziness, tremor, neuromuscular disease, gastroesophageal reflux , irritable bowel syndrome, cravings, gastric ulcer, gastrointestinal disorders, systemic weakness, face Edema, systemic edema, upper respiratory tract infection, rhinitis, sore throat, runny nose, irritation, depression, drowsiness, joint pain, flank pain, neck stiffness , back pain, BUN increase, bladder discomfort, cystitis, urolithiasis, hematuria, diabetes, urticaria, athlete's foot, Sweating, ringworm , hypotension, hypertension, cerebrovascular disease, peripheral vascular disease, flushing, nosebleeds, ALT increase, AST increase, viral infection, thyroiditis, and chronic myelogenous leukemia were 0.1% (1 patient, 1 case), respectively.

Among these, unexpected adverse drug reactions were heartburn , insomnia, and palpitations, respectively 0.7% (5 patients, 5 cases), abdominal pain 0.5% (4 people, 4 cases), urination , and blood glucose elevation 0.4% (3) People, 3 cases), neck / shoulder pain 0.3% (2 people, 3 cases), gastritis, chest pain, asthenia, lack of effect, weight loss, cough, cold, dry cough, anorexia, anxiety, back pain , blood Increased creatinine , ringing of 0.3% (2 people, 2 cases), dizziness, tremor, neuromuscular disease, gastroesophageal reflux , irritable bowel syndrome, cramping, gastric ulcer, gastrointestinal disorders, general weakness, generalized edema, upper respiratory tract infection, rhinitis, Sore throat, runny nose, irritation, drowsiness, joint pain, side pain, neck stiffness , back pain, BUN increase, bladder discomfort, cystitis, urolithiasis, hematuria, diabetes, urticaria, athlete's foot, sweating, ringworm , hypertension, cerebrovascular disease, peripheral Vascular diseases, flushing, nosebleeds, increased ALT, increased AST, viral infections, thyroiditis, and chronic myelogenous leukemia were reported in 0.1% (1 patient, 1 case), respectively.

-Significant drug adverse reactions include sensory abnormalities, chest pain, dyspnea, bradycardia , cerebrovascular disease, and chronic myelogenous leukemia, respectively, in 1 case, and chest pain, cerebrovascular disease, and chronic myelogenous leukemia are significant and expected. It was an adverse drug reaction.

4. General attention

The following are generally applied to beta-blockers.

1) Continuous use of beta-blockers reduces anesthetic induction and risk of arrhythmia during intubation . If the use of beta-blockers interferes with preparation for surgery, beta-blockers should be stopped at least 24 hours in advance . Anesthesia that causes myocardial suppression should be observed with caution .

   Vagus nerve reactions can be inhibited by intravenous administration of atropine .

2) Beta blockers can cause bradycardia, so if the pulse at rest drops to 50-55bpm or if it is determined to cause bradycardia, the dose is reduced.

3) Beta blockers can increase the sensitivity of the antigen and the degree of anaphylactic reaction.

4) For patients with ischemic heart disease , treatment with beta-blockers should be gradually stopped at intervals of 1 to 2 weeks or more. To prevent the angina from getting worse, other alternative treatments should be started at the same time.

5) Whether the patient's clinical condition (especially blood pressure, heart rate, conduction disorder, signs of deterioration of renal function), etc. should be observed under the supervision of an experienced physician for at least 2 hours after drug administration at each initial dose and dose escalation.

6) If the deterioration or intolerance of heart function is observed while increasing the drug , the first dose is administered or the dose is reduced and, if necessary, (severe hypotension, exacerbation of heart failure with acute pulmonary edema, shock of the heart , symptomatic The occurrence of bradycardia or atrioventricular blockage) Immediately stop drug administration.

5. Interaction

Pharmacodynamic interaction (common beta-blocker interaction)

: Drugs not recommended for administration

1) a calcium antagonist ( verapamil hydrochloride , dill thiazol jemgye a negative effect on), in combination with the shrinkable and atrioventricular upon administration iteulsu should note. Do not administer verapamil intravenously to patients treated with beta-blocker, as intravenous administration of verapamil to patients undergoing treatment with beta-blockers may result in severe hypotension and atrioventricular blockade .

2) Class 1 antiarrhythmic substances ( quinidine , dihydro quinidine , siben sleepy , play chi arsenide , diso pyramid , lidocaine , mexyl retinoic , Pro Pape rice ) and co-administration during the atrioventricular conduction time and negative for the byeonryeok action increases Concomitant use is not recommended.

3) The central action of antihypertensive drugs ( clonidine , Guan pasin , neck Sony Dean , methyl dopa , reel meni Din ) when administered in combination with the heart rate and the cardiac output of heart failure due to reduction of the central sympathetic nervous tension, such as reduction, vasodilatation Deterioration may occur. The risk of reactive hypertension may be increased by sudden discontinuation of this class of drugs (especially prior to discontinuation of beta blockers). Combination of the two drugs is not recommended.

: Drugs requiring careful attention to administration

4) The effect on AV loss time can be enhanced by co-administration with Class III antiarrhythmic drugs ( Amiodarone ) .

5) Combination use with anesthetics should reduce the reflex tachycardia and increase the risk of hypotension, so you should inform your anesthesiologist that you are receiving it.

6) When used in combination with insulin or oral diabetes medications , it does not affect blood sugar levels, but it can conceal certain symptoms of hypoglycemia (palpitations, tachycardia ) .

7) The antihypertensive drug may need to be adjusted because antihypertensive drugs may increase in blood pressure when administered in combination with anticonvulsant drugs ( baclofen ) or antitumor agents ( amiphostin ) and antihypertensive drugs.

: Drugs requiring attention

8) When combined with a beta-blocker , a digitalis agent can extend the AV conduction time . However, no clinical interactions were seen in the clinical trials of THIS DRUG and did not affect the pharmacodynamics of digoxin .

9) dihydropyridine type calcium antagonists ( amlodipine , felodipine , La CD pin , nicardipine , nimodipine , nitrendipine the risk of) the co-administration during blood pressure can increase it. In addition, the risk of worsening ventricular pump function in cardiac divers cannot be excluded.

10) Combination administration of antipsychotic and antidepressant drugs ( tricyclic , barbital , and phenothiazine ) can enhance the hypotensive effect of beta-blockers.

11) Combination use with NSAIDs does not affect the hypotensive effect of THIS DRUG.

12) Sympathetic neurostimulants can interfere with the effectiveness of beta-blockers.

Pharmacokinetic interaction

13) The drug is therefore metabolized by the CYP2D6 isozyme primarily metabolized through this route paroxetine , fluoxetine , Chi duck chopped , with quinidine increased blood levels of the drug by co-administration undue bradycardia and other adverse events Can increase the risk.

14) When combined with cimetidine, the blood concentration of THIS DRUG increases but the clinical effect remains unchanged. Concomitant administration with ranitidine does not affect the pharmacokinetics of this drug. This drug may be taken with food or when taking antacids between meals.

15) When combined with nicardipine, the blood concentration of the two drugs is slightly increased, but the clinical effect remains unchanged.

16) Co-administration with alcohol, furosemide or hydrochlorothiazide does not affect the pharmacokinetics of THIS DRUG .

17) This drug does not affect the pharmacokinetics or pharmacodynamics of warfarin .

6. Administration to pregnant and lactating women

1) The safety of administration during pregnancy has not been established. Beta blockers reduce placental reflux, which can lead to fetal death and premature and premature delivery in the womb. In addition, side effects ( hypoglycemia , bradycardia ) may occur in the fetus and newborn. The risk of heart and pulmonary complications in newborns is increased in the postnatal period. Therefore, this drug should not be given to pregnant women or pregnant women.

2) Most beta-blockers, especially fatty compounds, such as nebivolol and its active metabolites, pass through the milk. It is not known whether this drug is transferred to human breast milk, but it has not been administered during lactation as animal studies have reported a transition to breast milk .

7. Administration to children

Safety and efficacy have not been established for children under the age of 18, so it is not recommended to administer to children.

8. Administration to the elderly

Care is given to the elderly while observing the patient's condition, such as starting the administration at a low dose, paying attention to the following points.

1) In general, excessive blood pressure drop in elderly people is undesirable (a cerebral infarction may occur).

2) If a drug is needed, lose it slowly.

9. Treatment of overdose

1) Symptoms: When overdose, bradycardia , hypotension, bronchospasm, and acute heart failure may occur.

2) Treatment

① In the case of overdose or irritability, the patient is thoroughly protected and supervised and the blood glucose level is checked.

② Residual drug absorption in the gastrointestinal tract can be treated with gastric lavage , activated carbon, or administration of emollients.

③ Bradycardia or extensive vagus nerve reaction can be treated by administration of atropine or methyl atropine .

④ Low blood pressure or shock is treated with plasma or plasma substitutes , and catecholamines may be used if necessary.

⑤ β-blocking effect can be prevented by slow intravenous administration of an initial dose of about 5 ug / min of isoprenaline hydrochloride or 2.5 ug / min of dobutamine until the required effect is obtained . If refractory, isoprenaline may be administered with dopamine. If this does not work , consider intravenous administration of glucagon 50-100 ug / kg. If necessary, the injection is repeated within an hour, and if necessary, intravenous injection of glucagon 70 ug / kg / h can be added. Facemakers may be inserted to treat severely resistant bradycardia . End.

15.2 Herzog Ben sustained-release tablet PRECAUTIONS

1. Do not administer to the following patients.

1) People with severe congestive heart failure (which can worsen the symptoms of heart failure)

2) Patients with dysfunction syndrome, oriental block, atrioventricular block (2, 3 degrees) ( except for patients wearing artificial ventricular pacemakers )

3) Patients with hypotension (systolic pressure less than 90mmHg) or 쇽

4) Patients with a history of hypersensitivity to this drug

5) X-ray findings of acute myocardial infarction and pulmonary congestion Patients

6) Pregnant women or pregnant women

2. The next patient should be administered carefully.

1) Patients with congestive heart failure (which can worsen heart failure symptoms)

2) Patients with severe liver and kidney failure (drug metabolism and excretion may be delayed and action may be enhanced)

3) Patients with atrioventricular block (1 degree)

3. Side effects

1) When the daily dose of this drug is administered up to 540mg, the most common side effects are rhinitis, headache, sore throat, constipation, increased cough, influenza symptoms, peripheral edema, myalgia , diarrhea, vomiting, sinusitis , helplessness, low back pain, nausea, Indigestion, vasodilation, accidental injuries, abdominal pain, joints, insomnia, apnea , bleeding and tinnitus.

2) Cardiovascular: AV block (Fig. 1), arrhythmia, bradycardia , orthostatic hypotension, tachycardia , facial pallor , flushing, and stop , Oriental block, palpitations , ECG abnormalities, ST rise, chest pain , edema, congestive heart failure, rare complete atrioventricular block , significant bradycardia when the abnormality is recognized it may appear, such as, stop the administration, and sulfuric acid, atropine , isoproterenol and the appropriate treatment such as the heart rate as needed, together with the administration of such.

3) Mental nervous system: boredom, double feeling , calf cramps, helplessness, hypertension , sensory abnormalities, dizziness, confusion , headache may appear.

4) Digestive system: cravings, loss of appetite, abnormal teeth, belching, stomach discomfort , and heartburn .

5) Skin: sweating, skin hypertrophy, skin mucosal syndrome (Stevens-Johnson syndrome), addictive epidermal necrosis (Lyell syndrome) may appear.

6) Respiratory system: nasal bleeding , bronchitis, respiratory failure may occur.

7) Urinary system: cystitis, kidney stones , erectile dysfunction, amenorrhea, vaginitis, prostate disease may appear.

8) Metabolic and nutritional disorders: Gout and edema may appear.

9) Musculoskeletal system: Joint pain, mucositis , skeletal pain may appear.

1 0) Blood and lymphatic system : Lymphoma may appear.

11) systemic symptoms: pain, reaction, the causality is lacking barrel , neck stiffness , fever can occur.

12) Sensory organs: Amblyopia and earache may appear.

13) Hypersensitivity: Do not administer in this case because rash, itchiness, and rarely photosensitivity , polymorphic erythema erythema , and hives may appear.

14) Soy sauce: In rare cases, jaundice and hepatomegaly may appear. In addition, sometimes ALT and AST may be elevated.

15) Others: Gynecomastia, Parkinson's syndrome , platelet reduction, and leukocyte reduction may occur. In addition, gingival thickening may occur in rare cases depending on age .

4. General attention

1) Because the symptoms if you suddenly stop the administration of calcium antagonists could be worse for Withdrawal If I have enough observations while weight slowly.

2) ECG: This drug prolongs the recovery period of atrioventricular nodules without significantly delaying the recovery time of the nodules, except for patients with dysfunction syndrome . This effect can rarely cause abnormal bradycardia (especially patients with dysfunction syndrome), and sometimes atrioventricular block (2, 3 degrees).

3) Congestive heart failure: This drug has a negative effect on cardiac function or contractile force ( dP / dT) in the study of blood dynamics in the human body with normal ventricular function even though it exhibits negative muscle degeneration in animal tissues. Not shown. Acute studies of oral administration to patients with left ventricular dysfunction ( extraction coefficient 24 ± 6%) showed an increase in ventricular function coefficients without any particular reduction in contractile function. Exacerbation of congestive heart failure has been reported in patients with impaired ventricular function . Ventricular dysfunction patients to diltiazem and the β- blockers are administered in combination , because it is limited and caution when co-administration.

4) Hypotension: Reduction in blood pressure due to the administration of THIS DRUG can sometimes cause symptomatic hypotension.

5) As this drug is mostly metabolized in the liver and excreted through the kidneys and bile, regular observation is necessary if it is continuously administered in combination with other drugs . Be especially careful when administering to patients with liver and kidney failure. In the subacute / chronic toxicity test (dog, rat ), liver damage was observed when administered at high doses .

6) This drug is a sustained-release matrix. It has been reported that patients with severe stenosis have not reported obstruction, but should be used in patients with severe gastrointestinal stenosis.

7) Because dizziness may appear due to the effect of lowering blood pressure, care must be taken when operating machinery at high altitudes or operating a machine with dangers such as driving a car.

5. Interaction

1) Do not use terpenadin in combination with other antiarrhythmic drugs ( disopyramide phosphate ) and terpenadin as it has been reported to cause QT prolongation and ventricular arrhythmia . In addition, the drug and Ars temi sol QT prolongation, when administered in combination ventricular they may cause arrhythmia not administered in combination does.

2) Dantrolene ( injection solution ): Do not use together because ventricular fibrillation may occur .

3) Blood pressure lowering agents, nitrate preparations: Since it can enhance the blood pressure lowering effect, it is administered with caution.

4) β- blocker, la depression Piaget claim , arrhythmia solvent ( amiodarone , etc. ): bradycardia is to carefully dose can appear.

5) Digitalis preparations ( Digoxin , methyl digoxin ): Since it can increase the blood concentration of digitalis preparations, it is administered with caution.

6) Afrindine : The blood concentrations of the two drugs can be raised to each other, so it is administered carefully.

7) Cyclosporine : As it can increase the blood concentration of cyclosporine, it is administered with caution.

8) Rifampicin : This drug may reduce the action, so it is administered with caution.

9) Midazolam and phenytoin : Midazolam and phenytoin may be elevated in blood , so they should be administered with caution.

10) Cimetidine : This drug may increase the blood level, so be careful.

11) Theophylline : Theophylline metabolism and excretion may be delayed.

12) Anesthesia: Since it can enhance cardiac stimulation production and ECG suppression, it should be administered with caution.

13) Dihydropyridine-based calcium blockers ( nifedipine, etc. ): Dihydropyridine -based calcium blockers can be elevated in blood concentration, so it is administered with caution.

14) Carbamazepine : Increase the blood level of carbamazepine, so addiction symptoms (drowsiness, nausea, vomiting, dizziness, etc.) may occur, so be careful.

15) Tacrolimus : Since it can increase the blood concentration of tacrolimus, it is administered carefully.

16) Triazolam : Because triazolam can increase blood levels, it is administered with caution.

6. Administration to pregnant and lactating women

1) Animal experiments (mouse, rat , rabbit) showed a tendency to deform in the skeleton, heart, retina, and tongue. Also, weight loss and decrease in survival, delay in delivery, and increase in stillbirth were reported in the birth. Do not administer to pregnant or pregnant women.

2) Since the transfer of this drug to breast milk has been reported , avoid administration during lactation and discontinue lactation if unavoidable.

7. Administration to children

Safety and effectiveness in children have not been established.

8. Treatment of overdose

The initial treatment when overdose the token administered sikina induce vomiting and administration of activated charcoal to reduce the absorption of the drug. In addition to gastric lavage, the following methods can also be considered.

1) Bradycardia : Atropine (0.6-1.0 mg) is administered. If the vagus nerve is not blocked , isoproterenol is administered with caution .

2) High level atrioventricular block : Treat in the same way as the above bradycardia . If a high level atrioventricular block is fixed, it should be treated with a heartbeat .

3) Heart failure: Combined administration of a muscle-deficient drug (dopamine or dobutamine ) and a diuretic . It can be applied to administration of strong cardiac agents, blood pressure increasing agents, fluids, and auxiliary circulation.

4) Hypotension: Vasoconstrictor (dopamine or norepinephrine stannate ) is administered. It can be applied to administration of strong cardiac agents, blood pressure increasing agents, fluids, and auxiliary circulation. The actual treatment and dosage will depend on your physician's judgment, experience, and degree of clinical condition. Due to extensive metabolism, this drug is considered to be more than 10 times larger in blood concentration after administration of the upper dose, so be cautious when diagnosing overdose . Blood reflux of activated carbon is used as an adjuvant therapy to promote drug excretion .

5) There have been reports of successful treatment with appropriate treatment with activated carbon when the drug was orally administered in excess of 10.8 g.

9. Precautions for application (limited to capsules)

Be careful not to open or chew the capsules when taking this drug.

**16. Plan Violation, Suspension and Dropout Criteria**

16.1 Violation of the Plan

Clinical trials if the plan violated during the proceeding occurs, the test shall as soon as possible to sponsor the breach and inform , testing whether that person should continue the trial, must decide whether to abort. If you drop out of the course due to a violation of the plan, you should record it in the case report .

16. 2 Suspension and elimination criteria

- When a test subject who does not meet the selection / exclusion criteria participates, and when the selection / exclusion criteria are satisfied, but a violation of the plan has been confirmed.
- When the subject withdraws the consent to participate in the clinical trial
- If the subject cannot be tracked
- Serious adverse drug-related adverse reactions and side effects in the subjects
- If it is judged that it is impossible to proceed with the necessary examination or clinical trial for other reasons

**17. Evaluation variables**

17.1 Primary efficacy endpoint

( 1) msSBP and ms DBP changes at week 12 compared to baseline

( 2) baseline compared to 12 weeks of angina quality of life change

17.2 Secondary efficacy endpoints

( 1) baseline compared to 12 weeks of coronary spasm variation

Performing one more coronary angiography to assess vasospasm at week 12 is not very common in vasospastic angina patients, but evaluating the degree of vasospasm improvement if the patient agrees will help control the patient's medication And can be done at no additional cost associated with the inspection.

( 2) the baseline contrast six weeks msSBP and msDBP variation

( 3) the baseline contrast 6 weeks , 12 ratio has reached the target blood pressure at point

( 4) msSBP and msDBP changes at week 12 compared to week 2

( Target blood pressure *: msSBP / DBP <140 / 90mmHg)

17.3 Safety evaluation variables

Adverse reactions , laboratory tests , physical tests , vital signs ( pulse ), ECG

**18. Evaluation standards, evaluation methods and interpretation methods (statistical analysis methods)**

18.1 General principles of statistical analysis

Data obtained from subjects in this clinical trial are largely divided into FAS (Full Analysis Set) and PP (Per Protocol). Note analysis (main analysis) is the FAS, and further analysis of the clinical PP is to be analyzed. When the results of the two analyzes are compared and the results are different, the results of each method are presented and the reason is described in detail. All statistical tests are based on two-sided tests at a significance level of 5%.

18. 2 Analysis group definition

18.2.1 FAS (Full Analysis Set)

After randomization , subjects who have received at least one clinical trial drug and at least one efficacy endpoint after application are included in the analysis.

18.2.2 PP (Per Protocol)

PP is in accordance with the test plan clinical trials of subjects included in the FAS analysis in the clinical trials, clinical trials means the group is complete and the following if the test subjects except.

1) Subjects who drop out of the clinical trial and fail to meet the period specified in the study plan

2) Subjects who have received combination prohibition medication and combination prohibition therapy

3) Subjects who violate the selection / exclusion criteria

4) When it can be considered as a violation of other serious plans

18. 3 Missing value correction method

Validation at some point in the evaluation variable missing data is generated or clinical trials before the end of the test person is eliminated by missing this occurs the missing value data (available data, which does not replace analyzes to accept the set).

18. 4 Interim Analysis

No plans.

18. 5 Primary endpoint

18. 5 .1 msSBP and m s DBP changes at week 12 compared to baseline

For msSBP and msDBP , descriptive statistics (number of observations , mean , standard deviation , median , minimum , and maximum ) for baseline and 12- week outcome changes are presented for each treatment group, and comparison between the three groups for average change is ANOVA. Or analyze using Kruskal-Wallis test . Changes within each group are analyzed by paired t-test or Wilcoxon signed rank test. When there are more factors influencing , ANCOVA analysis is performed.

18. 5 .2 at week 12 compared to baseline quality of life angina change

Compare the average of the total scores combined through the baseline and the Seattle Angina Questionnaire at Week 12 . The comparison between the three groups for the mean change was analyzed using ANOVA or Kruskal-Wallis test . Changes within each group are analyzed by paired t-test or Wilcoxon signed rank test. When there are more factors influencing , ANCOVA analysis is performed.

18. 6 Secondary endpoint

18. 6 .1 week 12 compared to baseline coronary spasm variation

Coronary in spasm baseline and about 12 for the amount of change of the main results descriptive statistics ( number of observations subject , mean , standard deviation , median , minimum , maximum ) for each treatment group suggested and , the average of the change in the three groups of comparison ANOVA or Analyze using the Kruskal-Wallis test . Changes within each group are analyzed by paired t-test or Wilcoxon signed rank test. When there are more factors influencing , ANCOVA analysis is performed.

18. 6 .2 msSBP and msDBP changes at week 6 compared to baseline

For msSBP and msDBP , descriptive statistics (baseline, number of observations, mean, standard deviation, median, minimum, and maximum) for the change in baseline and 6-week results are presented for each treatment group, and the comparison between the three groups for the average change is ANOVA. Or analyze using Kruskal-Wallis test . Changes within each group are analyzed by paired t-test or Wilcoxon signed rank test. When there are more factors influencing , ANCOVA analysis is performed.

18. 6 .3 The percentage of target blood pressure reached at 6 and 12 weeks compared to baseline

Descriptive statistics (frequency and percentage) are presented for each treatment group for the rate at which target blood pressure was reached at 6 and 12 weeks compared to baseline , and the homogeneity between the three groups is analyzed using Pearson's chi-square test or Fisher's exact test. .

18. 6 .4 2 weeks compared to 12 weeks of msSBP and msDBP variation

For msSBP and msDBP , descriptive statistics (number of observations, mean, standard deviation, median, minimum, and maximum) for changes in the results of 2 weeks and 12 weeks are presented for each treatment group, and the comparison between the three groups for the average change is ANOVA. Or analyze using Kruskal-Wallis test . Changes within each group are analyzed by paired t-test or Wilcoxon signed rank test. When there are more factors influencing , ANCOVA analysis is performed.

**1 9 . Safety evaluation criteria including side effects , evaluation method and reporting method**

Assessment of adverse reactions will be evaluated and recorded at each visit during the 3-month study period, and will be conducted through interviews with patients and direct questions. Evaluation of safety is achieved through data collection of adverse reactions (frequency of adverse reactions, severity, types of side effects, serious adverse reactions, combined drugs, physical examination results, vital signs, electrocardiogram and blood tests, etc.) The symptoms and signs that the patient complains of will also be collected and recorded during the study period. If an adverse reaction occurs during a clinical trial, the researcher's judgment will decide whether to discontinue or continue the trial, and follow-up monitoring will be conducted until the result of the adverse reaction is resolved.

1 9 .1 Definition of adverse reactions

An 'adverse event (AE)' refers to any adverse and unintended symptoms (including signs, abnormalities in laboratory test results, etc.), symptoms, or diseases that occur in a subject who has been administered an investigational drug . That is, it is not necessary to have a causal relationship with the drug for the clinical trial.

'Adverse Drug Reaction (ADR)' refers to a case in which a causal relationship with a drug for clinical trial cannot be denied as any adverse and unintended reaction that occurred at any dose of drug used in a clinical trial.

'Serious AE / SAE' refers to any of the following cases among adverse reactions occurring at any dose of the drug used in clinical trials.

              -In case of death or danger to life

              -If you need to be hospitalized or need to extend your hospital stay

              -In case of permanent or serious disability and deterioration of function

              -When an abnormality or abnormality occurs in the fetus

'Least unexpected drug reactions (Unexpected Adverse Drug Reaction)' What is the clinical investigator or the kit aspects of adverse drug reactions or drug-related in the light of the available information, including attachments of drugs in to say that the difference in level I.

1 9 .2 Safety evaluation target

It is limited to subjects who have taken at least one drug for clinical trials.

1 9 .3 Evaluation criteria and methods

It is evaluated by taking into account the abnormalities in the results of laboratory test, vital signs of blood pressure and pulse, etc.

If there is clinical significance among abnormalities in laboratory tests, vital signs and abnormalities in physical examination, record them in the adverse reaction record sheet of the case report.

1 9 .3.1 Evaluation of adverse events

The case report form records the identified items such as the occurrence of adverse reactions, symptoms, date of expression, results, date of loss, severity, relationship with the drug for clinical trial, related measures, and treatment .

19.3.1.1 Criteria for the extent of clinical adverse events

① Mild: Subjects feel unpleasant to the extent that they do not limit their daily activities

② moderate (moderate): enough to limit their daily activities or affect the test subject feels discomfort

③ Severe: Adverse reactions that make everyday activities impossible

19.3.1.2 Relevance to clinical trial drugs

The investigator judges the relationship between the adverse reaction that occurred and the drug for clinical trials by reviewing the subject's past history, health status, dosing time, and dose status.

① Relevance is obvious

-If there is evidence that this drug has been administered

-When the time sequence of administration of this drug and the occurrence of adverse reactions is valid

-Adverse events are most likely explained by the administration of THIS DRUG than for any other reason

-When the adverse reaction disappears due to discontinuation of administration

- re-administered ( rechallenge embodiment, only when possible) if the result is positive,

-The adverse reaction is consistent with information already known about this drug or this class of drugs.

② A lot of relevance (probable / likely)

-If there is evidence that this drug has been administered

-When the time sequence of administration of this drug and the occurrence of adverse reactions is valid

-Adverse events are more probable by the administration of THIS DRUG than other reasons

-When the adverse reaction disappears due to discontinuation of administration

③ Suspicious (possible)

-If there is evidence that this drug has been administered

-When the time sequence of administration of THIS DRUG and adverse reactions is valid

-If it is judged that the administration of this drug is due to an adverse reaction at the same level as other possible causes

-When the adverse reaction disappears due to discontinuation of administration (if performed)

④ Little relevance (unlikely)

-If there is evidence that this drug has been administered

-If there are other possible causes for the adverse reaction

-If the result of discontinuation (if performed) is negative or ambiguous

- re-administered ( rechallenge embodiment, only when possible) if the result is negative or ambiguous

⑤ Not relevant (none)

-If the test subject did not receive this drug, or

-If the time sequence between drug administration and adverse reactions is not valid, or

-If there are other obvious causes for adverse reactions

⑥ evaluation impossible ( unassessable )

-When there is some information on adverse reactions, but it is not possible to evaluate the relationship with this drug

19.3.1.3 Actions related to clinical trial drugs

① Dose increase

② No dose change

③ Loss (dose reduced)

④ Dose interrupted

⑤ permanent withdrawal

⑥ Not applicable

⑦ unknown

19.3.1.4 Drug treatment

① Drug treatment for adverse reactions.

② No drug treatment was performed for adverse reactions.

19.3.1.5 Results

① Recovered / resolved

② Recovering / resolving

③ Not recovered / not resolved

④ Recovered (resolved) but sequelae remained (recovered / resolved with sequelae)

⑤ death

⑥ unknown (lost to follow up / unknown)

1 9 .4 How to report adverse events

The duties of each person in charge of 'significant adverse reactions' that occurred during the clinical trial period are as follows.

19.4. 1 Obligation of the person in charge of clinical trials

The clinical investigator should immediately report to the sponsor and support institution of any serious adverse events during the clinical trial (1 business day from the date of the investigator's knowledge) and report further with detailed information later. . However, if unexpected serious adverse drug reactions in clinical trials commission shall be reported promptly to the parties and the trial jury. In case of reporting an example of death, the investigator should provide additional information such as an autopsy report (only in the case of an autopsy) and a death certificate to the sponsor and the review committee.

19.4.2 Duties of Clinical Trials

If a serious adverse reaction occurs during a clinical trial, the person in charge of the clinical trial should immediately report it to the person in charge of the clinical trial and report further with detailed information later. However, unexpected unexpected adverse drug reactions should be promptly reported to the clinical trial sponsor and the clinical trial review committee.

19.4.3 Duties of the Institutional Review Board

The Investigation Committee shall take necessary measures to the responsible person of the clinical trial in the event of any unexpected or serious adverse drug reactions or new information that may adversely affect the safety of the subjects or the conduct of the clinical trial.

04/04/19 clinical trials of sponsor obligations

(1) the sponsor has (only if you need to change the test person in charge or did not report to the audit committee report applicable) reporting any serious adverse reactions and other related testers, the Jury received and the Food and Drug wife learned receive reports on The report must be made within 15 days of the date. However, in case of death or life-threatening, the fact must be reported within 7 days from the date of receiving or knowing, and in this case, detailed information shall be reported within 8 days from the initial reporting date. When the sponsor submits an adverse drug reaction report, he / she shall submit the information received from the person in charge of the clinical trial or the person in charge.

(2) The sponsor shall report additional safety information periodically in connection with the above reports until the corresponding adverse reaction is terminated (loss of the adverse reaction or no follow-up investigation is possible). At this time, the investigator should cooperate actively in providing data and information about the report.

(3) The sponsor support agency Korea menari you ㈜ of it to anyone looking person.

Contact information for clinical trial sponsors

And trying Guro Hospital, University Park, Chang - Kyu

Address: Cardiovascular Center, Guro-dong Hospital, 97, Guro-dong-gil, Guro-gu, Seoul

Phone: 02) 2626-3019

Support Organization Contact

Menarini Korea Co. , Ltd. , PV Manager Yeon- Woo Lee

Address: 12th floor, Seongdam Building, 411 Teheran - ro, Gangnam-gu, Seoul

Phone: 02) 2037-7393

Fax: 02) 2037-7373

Email: yeonwoo.lee@menariniapac.com

19 .5 Adverse reaction analysis method

Group -specific adverse events, adverse drug reactions (ADR), serious adverse events (SAE) occurred after drug administration with respect to each group descriptive statistics (expressed subjects can, expression suggests a 95% confidence interval for and expression cases) and incidence and treatment The difference in expression rates between groups is compared and analyzed using Pearson's chi-square test or Fisher's exact test.

Other laboratory test values and vital signs , such as with respect to when the resultant value is continuous variables drugs before administration compared to drug administration after 12 weeks for the amount of change for each treatment group descriptive statistics (number of observations subject, mean, standard deviation, median, minimum, maximum) of Present and analyze using ANOVA or Kruskal-Wallis test. Changes within each group are analyzed by paired t-test or Wilcoxon signed rank test. The result is the case of categorical variables, presenting a partition table for the drug before and after the change and gunnae change McNemar ' s analysis using the test and , groups differences Pearson ' s chi-square test or Fish er ' a s exact test Analysis.

**20. Standards of treatment and treatment of subjects after clinical trials**

After the trial for the subjects who have completed the clinical trial (including early termination), the treatment and treatment follow the routine treatment and treatment standards and principles.

**21. Measures to protect the safety of test subjects**

This study is conducted according to the clinical trial plan approved by the Clinical Trial Review Committee / Institutional Bioethics Committee and thoroughly reviewed and approved by the ethical and legal requirements of the study, and is also the basis of the clinical trial management standards and Helsinki Declaration throughout the entire course of the trial. You will be obedient. In the event of human rights violations in the study subjects during this study, the Institutional Review Board / Institutional Bioethics Committee will be notified.

Information that can identify the subject will be kept confidential by the researcher, and research data will be recorded with initials and coded subject identification information. In addition, subjects and study information will be stored on a computer with limited access and will be described and obtained consent in a separate space to protect subjects' identities. Even when the results of a clinical trial are published, the subject's personal information will remain confidential.

The researchers present the clinical trials involved subjects are clinical trial participation After serious adverse occurrence in such an emergency room visit or a researcher in the judgment in accordance with that emergency situation will determine if immediate clinical trials discontinued and necessary medical treatment should be . In addition , researchers in the judgment in accordance with the patient's abnormal condition is resolved or stabilized or more over medically that aid not need do until the track to observe and

**22. Other necessary matters to safely and scientifically conduct clinical trials**

22.1 Subject consent

Investigators should give patients and their caregivers sufficient opportunity to elaborate on all aspects of this trial and to know all predictable outcomes. The patient's consent must be documented.

The investigator must confirm by signing the subject consent form. Investigators should not perform specific tests intended solely for clinical trials until consent is obtained from the patient.

22.2 Confidentiality

Keep the names of all subjects confidential and identify subjects when recording and evaluating by the number assigned in the clinical trial . Subjects are informed that all clinical trial data is strictly confidential. The signed consent form is retained by the investigator. By signing this plan, the investigator agrees to obtain consent from the subjects who participate in the clinical trial and agrees to receive due diligence upon request. The head of the clinical trial maintains a list of the subject number and subject name so that records can be retrieved later. The consent form and list of subjects should be kept for 3 years from the date of completion of the clinical trial.

22.3 Familiarity with the clinical trial plan

The test manager and managers accurately analyze and understand the clinical trial plan and conduct the clinical trial.

22.4 Modification of clinical trial protocols

The contents of this study protocol cannot be altered during the study without the consent of the person in charge of the clinical trial. Once the test has started, corrections should be made only in exceptional cases. Any changes to the plan must be signed and agreed in writing by all parties involved. Any revised content must be approved by the Institutional Review Board.

22.5 Case Report Form (CRF)

Supporting documents referred to (source documents) refers to the patient records of doctors are kept in laboratory. Most supporting documents are charts of hospitals or physicians, and all information recorded in the patient's case record must match the supporting documents.

The data input and modification is done by the research personnel who have been delegated authority from the research director, and the final review and signature of the data is done by the research director. Through the signature, the research director guarantees that the information recorded in the case record is true, and in all cases, has the final responsibility for the accuracy and reliability of the information recorded in the case record.

In the case of amendments to the case record, the original record must be made available and the revised clinical investigator's signature must be entered.

22. 6 Monitoring and inspection

Monitoring and inspection can be conducted under the supervision of the supervisor of the research in order to ensure that clinical trials are conducted in accordance with KGCP and that test data are recognized at home and abroad.

As a data safety checker for this clinical trial , to ensure that the case records are complete and clear under the supervision of the research director , confirm that the research data has been collected in the case records according to the research plan and check with the records to ensure the completeness of the data. Review the safety data. The monitoring period should be carried out every year, at least upon initial registration.

22.7 Data Collection and Management

22.7.1 Record of clinical trial results

All data collected during this clinical trial should be recorded in the case record and the originals kept.

The researcher immediately records the data of the subject and clinical test results in the case report.

In case of missing data, the researcher should attach a reasonable explanation.

The completed case report is final signed by the research director.

22.7.2 Clinical Trial Data Management

Development, maintenance and data management of case records used in this clinical trial are performed by the sponsor . The input and management of clinical data recorded in the case report are managed by a separate person designated by the sponsor, and the sponsor must finally manage the completeness, accuracy and consistency of the data . When input of clinical data is completed, data check is performed to ensure the completeness , accuracy and reliability of the data . Clinical trials database used for data management is data loss due to system failure or disaster such as to prevent conduct regular backups to be managed so that recovery is possible.

22.7.3 Use of clinical trial results

Trial by signing this clinical protocol Dame representative will agree to use for the purpose of providing information for registration, presentation and professional uiyakhak The results of this test. Before publishing the results of this trial in an academic journal or journal, the sponsor of the trial has the right to review the presentation.

22.7.4 Storage of data

The original case report and all data and documentation related to the study should be retained for 3 years from the end of the clinical trial.

**23. References**

1. Schachinger V, Britten MB, Zeiher AM. Prognostic impact of coronary vasodilator dysfunction on adverse long-term outcome of coronary heart disease. Circulation. 2000; 101: 1899 -1906

2. Lerman A, Zeiher AM. Endothelial function: Cardiac events. Circulation. 2005; 111: 363 -368

3. Panza JA, Quyyumi AA, Brush JE, Jr., Epstein SE. Abnormal endothelium-dependent vascular relaxation in patients with essential hypertension. The New England journal of medicine. 1990; 323: 22 -27

4. Taddei S, Virdis Amps, Mattei P, Salvetti A. vasodilation to acetylcholine in Primary and Secondary Forms of the Human hypertension. Hypertension. 1993; 21: 929 -933

5.Rees DD, Palmer RM, Moncada S. Role of endothelium-derived nitric oxide in the regulation of blood pressure. Proceedings of the National Academy of Sciences of the United States of America. 1989; 86: 3375 -3378

6. Suwaidi JA, Hamasaki S, Higano ST, Nishimura RA, Holmes DR, Jr., Lerman A. Long-term follow-up of patients with mild coronary artery disease and endothelial dysfunction. Circulation. 2000; 101: 948 -954

7. Yasue H, Omote S, Takizawa A, Nagao M. Coronary arterial spasm in ischemic heart disease and its pathogenesis. A review. Circulation research. 1983; 52: I 147-152

8. Cockcroft JR, Chowienczyk PJ, Brett SE, Chen CP, Dupont AG, Van Nueten L, Wooding SJ, Ritter JM. Nebivolol vasodilates human forearm vasculature: Evidence for an l-arginine / no-dependent mechanism. The Journal of pharmacology and experimental therapeutics. 1995; 274: 1067 -1071

9. Tzemos N, Lim PO, MacDonald TM. Nebivolol reverses endothelial dysfunction in essential hypertension: A randomized, double-blind, crossover study. Circulation. 2001; 104: 511 -514

10. Dessy C, Saliez J, Ghisdal P, Daneau G, Lobysheva , II, Frerart F, Belge C, Jnaoui K, Noirhomme P, Feron O, Balligand JL. Endothelial beta3-adrenoreceptors mediate nitric oxide-dependent vasorelaxation of coronary microvessels in response to the third-generation beta-blocker nebivolol. Circulation. 2005; 112: 1198 -1205

11.Maffei A, Lembo G. Nitric oxide mech anisms of Nebivolol
